# A TtAgo‐Driven Autocatalytic Circuit with Thermal‐Enhanced Kinetics for One‐Pot Nucleic Acid Detection

**DOI:** 10.1002/advs.202521671

**Published:** 2025-12-12

**Authors:** Zuowei Xie, Ruijia Deng, Ben Niu, Yingjie Yang, Shuang Zhao, Hongzhao Yang, Meilin Gong, Jie Luo, Yu Tang, Jing Sheng, Yan Pi, Ming Chen, Kai Chang

**Affiliations:** ^1^ Department of Clinical Laboratory Medicine Southwest Hospital Third Military Medical University (Army Medical University) Chongqing 400038 China; ^2^ Department of Rehabilitation Medicine The First Affiliated Hospital of Chongqing Medical University Chongqing 400042 China; ^3^ State Key Laboratory of Trauma and Chemical Poisoning Army Medical University 30 Gaotanyan, Shapingba District Chongqing 400038 China

**Keywords:** argonaute, catalytic DNA circuits, machine learning, nucleic acid detection, rolling circle amplification

## Abstract

Catalytic DNA circuits hold significant promise for nucleic‐acid‐based diagnostics, yet they remain hindered by slow reaction kinetics and the non‐universal nature of one‐pot approaches. Here, a universal catalytic DNA circuits termed TACTIC (Thermus thermophilus Argonaute (TtAgo) protein‐driven autocatalytic circuit) is developed for one‐pot detection of DNA and RNA in multiple clinical samples. TACTIC employs the heat‐activated cleavage activity of TtAgo to accelerate reaction kinetics of rolling circle amplification (RCA) by producing an efficient circular template with Gibbs free energy approaching zero at 70. Combined with TtAgo cleavage‐mediated explosive regeneration and accumulation of target mimics, an autocatalytic positive‐feedback circuit is successfully constructed for universal and sensitive detection of nucleic acid biomarkers. Efficient TACTIC is developed by increasing 381% amplification efficiency and achieved detection sensitivity at the attomolar (aM) level. TACTIC enables rapid one‐pot detection of bacterial DNA, mutant mRNA, and four extracellular vesicle‐derived miRNAs (EV miRNAs) within 30 min. Integrated with machine learning, the distinct expression patterns of four EV miRNAs across different biofluids are accurately profiled, and machine learning‐driven diagnostic and staging models for breast cancer are established within a clinical cohort. TACTIC offers new insights for advancing higher‐order catalytic circuits and expanding the toolbox for accurate nucleic acid detection.

## Graphical abstract

This study proposes a universal catalytic DNA circuits termed TACTIC (Thermus thermophilus protein‐driven autocatalytic circuit) for one‐pot detection of DNA and RNA in multiple clinical samples. Integrated with machine learning, TACTIC accurately profile the distinct expression of four extracellular vesicle‐derived miRNAs across different samples and establish machine learning‐driven diagnostic and staging models for breast cancer within a clinical cohort.

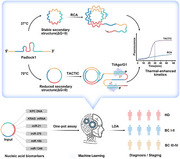

## 1 Introduction

Catalytic DNA circuits with dynamic responsiveness and programmability can perform diverse functions, including signal amplification,^[^ ^1^, ^2^ ^]^ logic operations,^[^ ^3^, ^4^ ^]^ and cascade reactions through the design of specific DNA sequences and reaction pathways,^[^ ^5^, ^6^ ^]^ which have garnered significant attention in biosensing and bioengineering. Various catalytic DNA circuits have been developed and applied in clinical diagnostics,^[^ ^7^ ^]^ live‐cell imaging,^[^ ^8^, ^9^ ^]^ and molecular computing.^[^ ^10^ ^]^ However, traditional catalytic DNA circuits, specifically enzyme‐free circuits, often suffer from low reaction rates,^[^ ^11^, ^12^ ^]^ which is highly unfavorable for the development of in vitro diagnostic technologies aiming for ultra‐high sensitivity. Furthermore, in most one‐pot assays based on catalytic DNA circuits, the universality of the method is severely compromised by inevitable background leakage and suboptimal cross‐reactivity,^[^ ^13^, ^14^, ^15^ ^]^ making multiplex detection for different targets significantly challenging. Consequently, there is a pressing need to develop novel catalytic circuits capable of achieving ultrasensitive responses to low‐abundance inputs, one‐to‐multiple cascading outputs efficiently, and reliable detection across diverse nucleic acid targets in a one‐pot assay.

Inspired by the Q_10_ rule in enzyme kinetics,^[^ ^16^ ^]^ elevating the reaction temperature of catalytic DNA circuits represents a feasible and efficient approach to accelerate reaction rates. This thermal‐enhanced kinetics effect improves molecular kinetic energy and reduces thermodynamic barriers while preserving circuit programmability. Argonaute (Ago) proteins, particularly thermophilic variants, inherently possess this effect due to their characteristic high‐temperature‐tolerant activity. As sequence‐specific endonucleases, Ago proteins exhibit robust nucleic acid recognition and cleavage capabilities.^[^ ^17^ ^]^ Compared to eukaryotic Ago proteins (eAgos), the more versatile prokaryotic Ago proteins (pAgos) utilize short DNA guides (gDNA) to bind complementary target nucleic acids via base pairing, enabling precise cleavage target between the 10th and 11th nucleotides from the 5' end of the gDNA.^[^ ^18^, ^19^, ^20^ ^]^ Notably, unlike the clustered regularly interspaced short palindromic repeats (CRISPR)/CRISPR‐associated protein (Cas) systems,^[^ ^21^, ^22^ ^]^ which also offer sequence‐specific recognition and efficient catalytic cleavage, Ago‐mediated cleavage operates independently of protospacer adjacent motif (PAM) sequences.^[^ ^23^ ^]^ Furthermore, gDNA offers superior stability and cost‐effectiveness compared to RNA guides (crRNA), providing greater flexibility in probe sequence design.^[^ ^24^ ^]^ These properties theoretically render Ago proteins more compatible with catalytic DNA circuits than CRISPR/Cas systems, offering a promising platform for constructing simpler yet comparably ultrasensitive autocatalytic feedback circuits.

Recently, an artificial nucleic acid circuit based on Ago proteins was proposed for one‐step isothermal detection of clinical drug‐resistant bacteria.^[^ ^25^ ^]^ Impressively, it represented an innovative attempt to synergistically integrate an artificial nucleic acid circuit with an Ago protein, leveraging the target recognition and cleavage activity of Ago to establish a positive feedback loop with high specificity and exponential signal amplification. However, the method's reliance on high Ago protein concentrations hindered the establishment of a dynamic equilibrium between non‐specific cleavage and efficient amplification. Coupled with prolonged reaction times, these limitations impeded its broad clinical diagnostic application. Other Ago‐based circuits incorporated pre‐amplification techniques such as loop‐mediated isothermal amplification (LAMP) or recombinase polymerase amplification (RPA) to boost low‐abundance nucleic acids prior to using Ago as a signal reporter.^[^ ^26^, ^27^, ^28^ ^]^ Nevertheless, these approaches decoupled amplification from detection. The complex handling involved increases the risks of enzymatic degradation, cross‐contamination, analytical variability, and false‐positive results, while also extending assay time.^[^ ^29^, ^30^ ^]^

Herein, we integrated Thermus thermophilus Argonaute protein (TtAgo) with rolling circle amplification (RCA) to construct a TtAgo‐driven autocatalytic positive feedback circuit (TACTIC) for the universal detection of nucleic acid biomarkers. To our knowledge, this represents the first report integrating the programmable sequence‐guided cleavage activity of an Ago protein with RCA. Compared to previously reported Ago‐based circuits and conventional RCA, TACTIC with thermal‐enhanced kinetics exhibits three distinct advancements. First, it establishes a viable strategy to balance reaction kinetics by coordinating TtAgo's endonuclease activity with DNA polymerase activity under precise temperature control. This dual‐enzyme system preserves TtAgo's cleavage efficiency while ensuring robust polymerization, enabling a streamlined one‐tube workflow that simplifies the experimental procedure and shortens detection time. Second, leveraging TtAgo's thermophilic nature, the reaction temperature is elevated to 70 °C (vs 37 °C in traditional RCA), which reduces duplex stability to minimize polymerase resistance and accelerate molecular diffusion to increase effective collision frequency, enhancing RCA amplification efficiency. Third, TtAgo continuously cleaves RCA amplicons, leading to the explosive regeneration and accumulation of target mimics, thereby establishing a positive feedback loop that significantly boosts detection sensitivity. We anticipate that TACTIC has significant application potential for point‐of‐care disease diagnosis.

## 2 Results

### 2.1 Study Principles of TtAgo‐Driven Autocatalytic Circuit

**Scheme 1:**
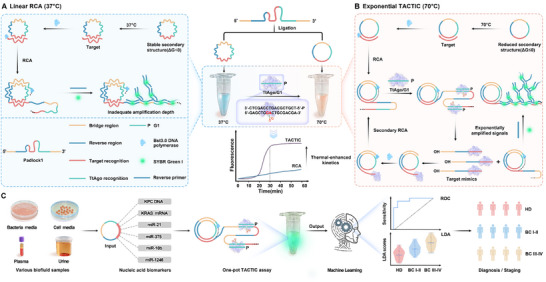
Flowchart of the TACTIC assay for one‐pot nucleic acid biomarker detection. A) Schematic diagram of conventional linear RCA. B) Schematic diagram of TtAgo‐driven RCA (TACTIC). C) Application of TACTIC for universal nucleic acid biomarker detection and diagnosis of breast cancer using the four EV miRNAs combined with machine learning analysis.

As shown in Scheme 1, before the assay, the linear padlock probe is synthesized into a circular template encoding multiple functional regions via traditional denaturation‐annealing‐ligation. This circular template comprises four modular sequences: a bridge region (yellow) ligating the 5′‐phosphate and 3′‐hydroxyl termini of the padlock 1 through complementary pairing with the bridge primer (BP); a reverse region (blue) triggering hyperbranched rolling circle amplification (HRCA) upon hybridization of its complementary product with the reverse primer (RP), enabling fluorescence signal output via dye binding; a target recognition region (red) specifically binding the target sequence to initiate RCA; a TtAgo recognition region (green) whose complementary sequence can be specifically recognized and cleaved by the TtAgo/gDNA1 (G1) complex. Within TACTIC, the introduction of the thermophilic TtAgo protein necessitates a higher reaction temperature. Consequently, compared to the circular template in conventional RCA (37 °C), which exhibits a stable secondary structure (ΔG < 0 kcal mol^−1^), the identical circular template in TACTIC achieves a Gibbs free energy change approaching zero (ΔG 0 kcal mol^−1^) at 70 °C due to effective disruption of its secondary structure. This reduction in structural stability minimizes polymerase progression resistance, thereby leading to the thermal‐enhanced reaction kinetics and amplification efficiency. Following the combination of the circular template with all other reagents into a single tube, the target is extended by Bst 3.0 DNA polymerase, generating long single‐stranded DNA (ssDNA) concatemers containing complementary copies of the circular template. As depicted in Module A, conventional RCA follows a linear concatemer amplification pathway, exhibiting inadequate amplification depth. In contrast, within the TACTIC system (module B), the TtAgo/G1 complex specifically binds to the TtAgo recognition sites on these concatemers and cleaves them, producing target mimics bearing 3′‐hydroxyl termini. These target mimics, containing complementary copies of the circular template, subsequently act as new primers to initiate a second round of RCA. This process ultimately establishes a self‐sustaining positive feedback loop, successfully converting the traditional linear RCA into an exponential amplification cascade, significantly improving detection sensitivity. In Module C, the universality and reliability of TACTIC are validated by detecting the Klebsiella pneumoniae carbapenemase (KPC) resistance gene, KRAS^G12D^ mRNA, and four extracellular vesicle‐derived miRNAs (EV miRNAs) through modular redesign of the padlock probe sequences. Machine learning‐assisted intelligent diagnosis is further implemented to profile four EV miRNAs across diverse biofluids and clinical cohorts, developing diagnostic and staging models for breast cancer (BC).

### 2.2 Feasibility Analysis of TACTIC Assay

**Figure 1:**
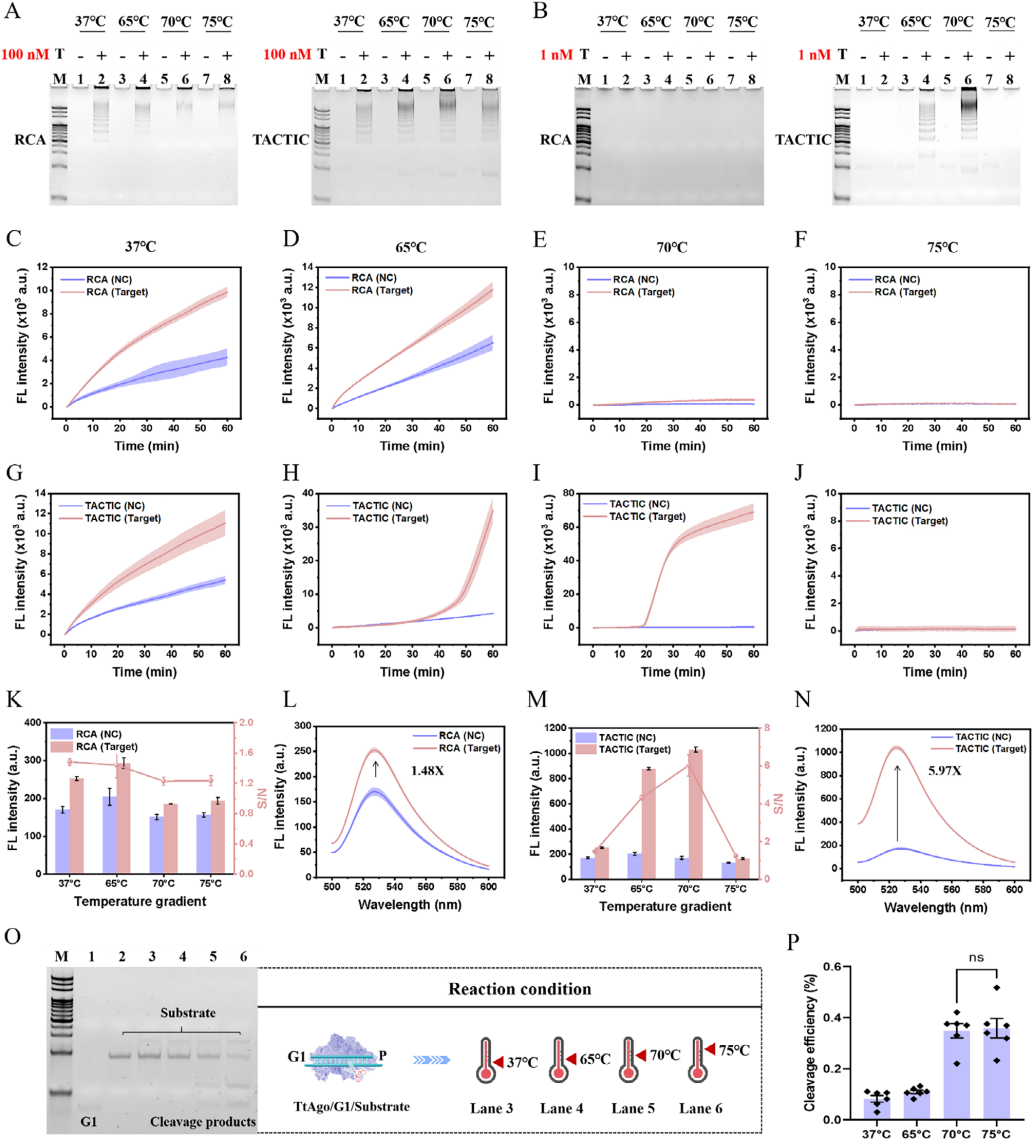
Feasibility analysis. A,B) PAGE analysis of the nucleic acid amplification capabilities of both RCA and TACTIC under four temperature gradients in the presence of high and low target concentrations, respectively. The four temperature gradients were set as 37, 65, 70, and 75 °C. C_miR‐21_ = 1 nm, C_padlock 1_ = 100 nm, C_TtAgo_ = 100 nm, C_G1_ = 25 nm, C_Bst__3.0_ = 0.1 U/µL. C–F) Real‐time fluorescence monitoring of RCA under the four temperature gradients. G–J) Real‐time fluorescence monitoring of TACTIC under the four temperature gradients. K) The signal‐to‐noise ratios of RCA under the four temperature gradients. L) The fluorescence spectrum of RCA at 37 °C. M) The signal‐to‐noise ratios of TACTIC under the four temperature gradients. N) The fluorescence spectrum of TACTIC at 70 °C. O) PAGE analysis of the DNA cleavage capacity of TtAgo protein under the four temperature gradients. C_Substrate_ = 100 nm, 1 h. P) The band intensities corresponding to Figure O were quantitatively analyzed using ImageJ software to evaluate the DNA cleavage efficiency of the TtAgo protein. The summed intensity of the as‐introduced intact substrate band (without TtAgo/G1) and as‐involved substrate (with TtAgo/G1) band were respectively set as the total and residual amount of substrate, and were respectively served as the minuend and subtrahend in the calculation of the cleaved substrate. Then the cleavage efficiency could be acquired by dividing the initially introduced substrate with the calculated cleaved substrate. Bars represent the mean ± SD (*n* = 6), ns indicates no statistical significance.

To validate the feasibility of the TACTIC assay, miR‐21 was selected as a model target. Initially, polyacrylamide gel electrophoresis (PAGE) was employed to evaluate the amplification capabilities of both conventional RCA and TACTIC across four temperature gradients. As shown in **Figure** **1**A, in the presence of a high target concentration (100 nm), both methods produced significant amplification bands compared to the negative control. When the target concentration was reduced to 1 nm (Figure 1B), no discernible amplification band was observed for conventional RCA relative to the negative control. Conversely, the TACTIC generated substantial amplification bands at reaction temperatures of 65 and 70 °C, with the band at 70 °C being notably brighter and sharper. These contrasting electrophoretic profiles confirmed that exponential TACTIC possesses superior nucleic acid amplification capability compared to traditional linear RCA.

Then, real‐time fluorescence monitoring was performed to track signal changes for both methods across the four temperatures. As depicted in Figure 1C–F, when the reaction temperature reached 70 ℃, target‐triggered RCA failed to generate distinguishable fluorescence signals, consistent with the fluorescence spectra results (Figure S1A–D, Supporting Information). Notably, TACTIC exhibited markedly distinct fluorescence signals at the identical 70 °C condition, with higher fluorescence intensity than observed at 37 or 65 °C (Figure 1G–J). However, as the temperature increased further, TACTIC also displayed a significant reduction in fluorescence signal, aligning with the fluorescence spectra data (Figure S1E–H, Supporting Information). This phenomenon can be attributed to the inhibitory effect of elevated temperature on DNA polymerase activity. Published reports indicate that Bst 3.0 DNA polymerase exhibits optimal activity at 65 °C, with a maximum operating temperature not exceeding 72 °C. Consequently, polymerase activity is progressively inhibited with increasing temperature, culminating in complete inactivation at 75 °C. Unlike the single‐enzyme RCA assay, the dual‐enzyme TACTIC achieved a dynamic equilibrium between the endonuclease activity of TtAgo and the polymerase activity of Bst 3.0 at 70 °C, resulting in significantly enhanced fluorescence intensity. Fluorescence spectra results demonstrated that conventional RCA achieved its optimal signal‐to‐noise ratio (SNR) of 1.48 at 37 °C, whereas TACTIC attained a substantially higher optimal SNR of 5.97 at 70 °C, further evidencing the superior analytical performance of TACTIC (Figure 1K–N).

We subsequently verified the cleavage capability of the TtAgo protein at 70 °C and comparing its activity with that at its optimal temperature of 75 °C. As shown in Figure 1O, 47‐nt amplification products was selected as the substrate for the TtAgo/G1 system, and its nuclease activity was assessed under four temperature conditions. Compared to the intact substrate band (Lane 2), two clear cleavage bands with faster electrophoretic mobility were observed in Lanes 5 and 6. The substrate band intensity (lane 3–6) was then quantified to calculate cleavage efficiency of TtAgo. As illustrated in Figure 1P, there was no statistically significant difference in the DNA cleavage efficiency of the TtAgo/G1 system at 70 ℃ compared with that at 75 ℃ (*p* > 0.05), indicating that reducing the reaction temperature to 70 °C did not significantly impair the DNA endonuclease activity of the TtAgo protein.

### 2.3 Thermal‐Enhanced Kinetics Studies

**Figure 2:**
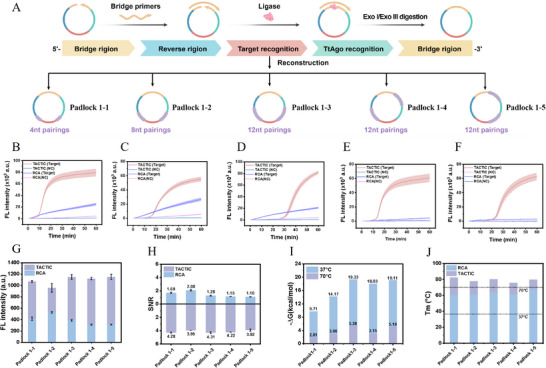
Evaluation of temperature effects on reaction kinetics and amplification efficiency. A) Schematic diagram of reconstruction of padlock probes. B–F) Real‐time fluorescence monitoring of RCA and TACTIC using five reconstructed padlock probes (Padlock1‐1, Padlock1‐2, Padlock1‐3, Padlock1‐4, Padlock1‐5), respectively. C_miR‐21_ = 1 nm, C_padlock_ = 100 nm, C_TtAgo_ = 100 nm, C_G1_ = 25 nm, C_Bst 3.0_ = 0.2 U/µL. G) Comparison of endpoint fluorescence intensities. H) Comparison of amplification signal‐to‐noise ratios. I) Predicted free energy of the five padlock probes at 37 and 70 °C. (J) Predicted melting temperature of the five padlock probes at 37 and 70 °C, respectively. Bars represent the mean ± SD (*n* = 3).

It is well‐documented that factors such as circular template size, sequence context, secondary structure, and topological features significantly impact RCA amplification efficiency.^[^ ^31^, ^32^, ^33^ ^]^ Consequently, designing more efficient circular templates by minimizing their secondary structure represents a viable strategy to enhance amplification efficiency. Compared to optimizing template sequence composition to reduce secondary structure, elevating the reaction temperature offers a simpler and more direct approach. Following the establishment of 70 °C as the optimal reaction temperature for TACTIC, we further investigated the influence of temperature on RCA amplification efficiency. **Figure** **2**A clearly illustrates the formation process of the padlock probe into the circular template and highlights the distinct functional regions on the probe. Successful synthesis and purification of the circular template were confirmed by PAGE (Figure S2, Supporting Information). Subsequently, we employed a reconstruction strategy, deliberately designing complementary sequences of varying lengths within different functional regions of the padlock probe (padlock 1–1 to 1–5). This design aimed to introduce more stable secondary structures and self‐dimers into the padlock probe. The amplification efficiency of conventional RCA was then compared to that of TACTIC using these reconstructed probes. As shown in Figure 2B–F, compared to the linear amplification profile of conventional RCA, TACTIC exhibited an exponential growth curve and generated substantially stronger fluorescence signals (Figure 2G). Additionally, under identical conditions, non‐specific amplification in TACTIC was robustly suppressed, resulting in a higher SNR (Figure 2H). This can be attributed to the elevated temperature weakening mismatch binding between primers and templates, thereby suppressing non‐specific amplification and reducing background noise.^[^ ^34^ ^]^

The secondary structure of nucleic acid probes is usually accurately assessed by changes in free energy. To verify the above conclusion, we used Mfold to predict the free energy and conformations of the nucleic acid probes at 37 and 70 °C, respectively (Figure S3, Supporting Information). As shown in Figure 2I, under the 70 °C condition, the free energies of the five reconstructed probes were closer to 0 kcal mol^−1^ compared to those at 37 °C, indicating that the formation of secondary structures is less likely. This further confirms that increasing the reaction temperature of traditional RCA can efficiently disrupt the secondary structures on the template, reduce polymerase amplification resistance, and ultimately significantly enhance reaction kinetics and amplification efficiency. Next, considering the risk of ring‐opening of the circular template at high temperatures, we evaluated the stability of the circular templates using the melting temperature (Tm) parameter. As shown in Figure 2J, the Tm values of the five reconstructed probes were all significantly higher than the actual reaction temperature, demonstrating that under high‐temperature conditions, the circular templates still maintain a stable spatial structure. Furthermore, the 12% PAGE experiments were conducted to further characterize the time‐dependent behavior of these circular templates at 70 °C, revealing no significant template degradation within the first hour (Figure S4, Supporting Information). In summary, our design effectively maintains a balance between temperature and template stability.

### 2.4 Endonucleases Screening and Workflow Optimization

**Figure 3:**
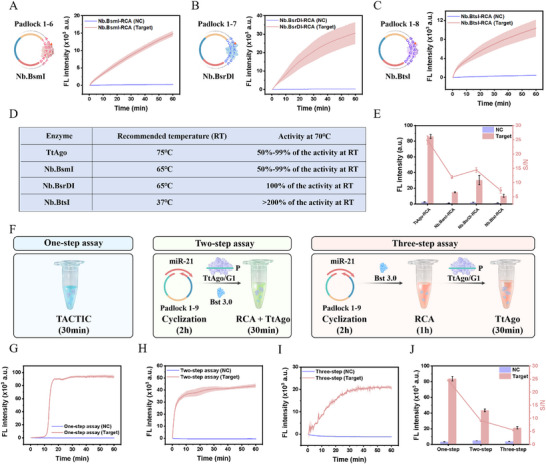
Endonucleases Screening and Workflow Optimization. A) Real‐time fluorescence monitoring of the redesigned padlock probe (Padlock 1–6) recognizable by Nb.BsmI. B) Real‐time fluorescence monitoring of the redesigned padlock probe (Padlock 1–7) recognizable by Nb.BsrDI. C) Real‐time fluorescence monitoring of the redesigned padlock probe (Padlock 1–8) recognizable by Nb.BtsI. D) Comparison of working temperatures and enzymatic activities of the four endonucleases. E) Comparison of the amplification capabilities of RCA mediated by the four endonucleases. F) Schematic illustration of the one‐step, two‐step, and three‐step TACTIC assays. G) Real‐time fluorescence monitoring of the one‐step TACTIC assay. H) Real‐time fluorescence monitoring of the two‐step TACTIC assay. I) Real‐time fluorescence monitoring of the three‐step TACTIC assay. J) Comparison of the analytical performance of the three workflows. Bars represent the mean ± SD (*n* = 3).

To highlight the critical role of TtAgo in our assay, we selected three common restriction endonucleases to replace TtAgo in mediating distinct RCA reactions at 70 °C (BsmI‐RCA, BsrDI‐RCA, BtsI‐RCA). Corresponding padlock probes (padlock 1–6 to 1–8) were designed based on the specific cleavage sites of these nucleases. As shown in **Figure** **3**A–C, all three nuclease‐mediated RCA assays produced significant fluorescence signal differences compared to the negative control. The selection of these nucleases was primarily based on their thermostability, enabling maintained good enzymatic activity at 70 °C (Figure 3D). In Figure 3E, while the three nuclease‐mediated RCA assays generated detectable signal differences, the TACTIC assay achieved a substantially higher SNR. This is attributed to TtAgo's unique thermophilic characteristics and highly efficient nuclease activity, which collectively confer superior signal amplification capability. Furthermore, we used three padlock probes to construct different circular templates with the TtAgo recognition region positioned at the right end (padlock 1), left end (padlock 2), and middle of the sequence (padlock 3), respectively, to investigate the impact of cleavage site location on amplification (Figure S5, Supporting Information). The results demonstrated that padlock 1 generated the strongest detection signal and achieved the highest SNR. We hypothesize that positioning the cutting site at the terminus of the template may minimize secondary structure formation, thereby reducing steric hindrance and facilitating optimal spatial binding and cleavage efficiency of the TtAgo/gDNA complex, which is consistent with the conclusion that elevated temperature reduces secondary structure.

Subsequently, to optimize the overall detection workflow, we artificially divided the one‐step TACTIC process into two‐step (target‐induced circularization and RCA/TtAgo cleavage) and three‐step (target‐induced circularization, RCA, then TtAgo cleavage) protocols (Figure 3F). As illustrated in Figure 3G–J, compared to the two‐step and three‐step assays, the one‐step assay, despite exhibiting a slightly reduced overall reaction speed (manifested as a delayed inflection point in the amplification curve), produced significantly enhanced detection signals and achieved the highest SNR. This is because in the single‐tube format, signal generation occurs concurrently with the amplification of low‐abundance targets. In contrast, in multi‐step formats, the subsequent signal generation reaction initiates only after the accumulation of substantial triggers from the preceding amplification step.^[^ ^35^ ^]^ Therefore, combining all reagents into a single tube for a one‐pot reaction not only significantly improves detection sensitivity but also drastically shortens assay time, eliminates repeated tube‐opening maneuvers, and reduces the risk of cross‐contamination, thereby enhancing result reliability.

### 2.5 Detection Performance Evaluation of TACTIC Assay

**Figure 4:**
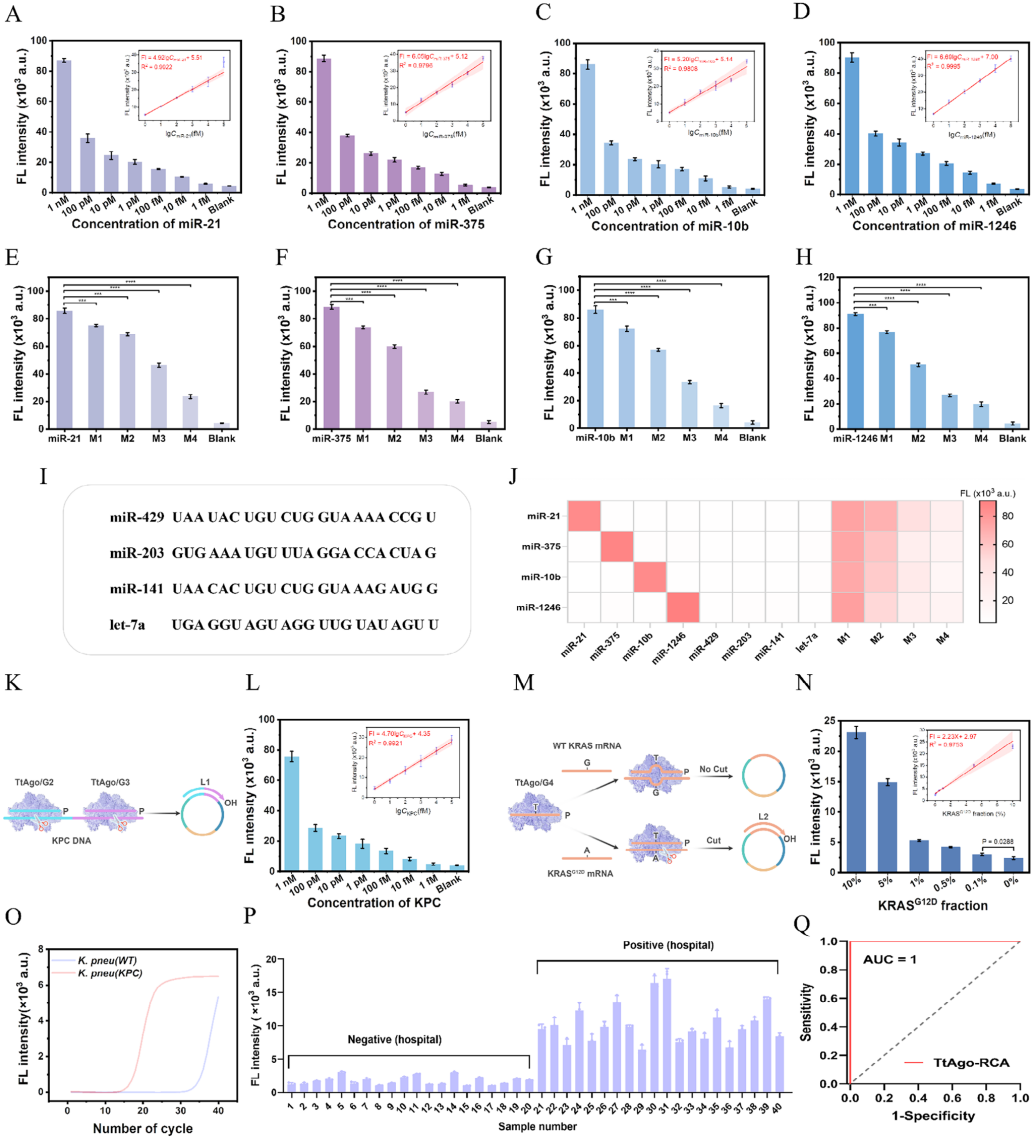
Detection performance evaluation of TACTIC. A–D) Sensitivity analysis targeting miR‐21, miR‐375, miR‐10b, and miR‐1246, respectively, with corresponding linear regression equations. The concentration gradients of the four miRNAs were set at 1 nm, 100 pm, 10 pm, 1 pm, 100 fm, 10 fm, and 1 fm. E–H) Single‐base mutation discrimination analysis targeting miR‐21, miR‐375, miR‐10b, and miR‐1246, respectively. Bars represent the mean ± SD (*n* = 3), ^*^ *p* < 0.05, ^**^ *p* < 0.01, ^***^ *p* < 0.001, ^****^ *p* < 0.0001. I) Detailed base sequences of miRNA homologs; J) Specificity heatmap of TACTIC. M1–M4 represent variants with different numbers of base mutations. The concentrations of the target miRNA and other miRNA homologs were maintained at 1 and 10 nm, respectively. K) Schematic illustration of the TACTIC assay for KPC DNA detection. L) Sensitivity analysis for KPC DNA with linear regression equation. M) Schematic illustration of the TACTIC assay for KRASG12D mRNA detection. N) Sensitivity analysis for KRAS^G12D^ mRNA with linear regression equation. O) qPCR validation of the resistance gene expression. P) Discrimination of CPKP and wild‐type KP by TACTIC (*n* = 20 clinical isolates each). Q) ROC analysis.

Key parameters of the TACTIC assay were first optimized to achieve optimal detection performance (Figure S6, Supporting Information). It is noteworthy that, given the signal continues to increase until reaching a plateau at 30 min, a detection time point of 30 min was selected as the standardized endpoint to achieve an optimal balance between sensitivity and assay speed. Subsequently, the sensitivity, specificity, and universality of the conventional RCA and TACTIC assays were systematically investigated and compared. As illustrated in **Figure** **4**A–D, four miRNAs (miR‐21, miR‐375, miR‐10b, miR‐1246) were specifically targeted and detected simply by reprogramming the target recognition region on the circular template. Serially diluted samples of the four miRNAs were prepared, and the fluorescence signals were collected using both RCA and TACTIC, respectively. A calibration curve was established by plotting fluorescence intensity against the logarithm of the target concentration to determine the limit of detection (LOD) and linear dynamic range. Within the range of 1 fM–100 pM, a strong linear relationship was observed between the logarithm of the miRNA concentration and the fluorescence intensity for TACTIC. Based on the 3σ/slope criterion, TACTIC achieved LODs of 37.5 am for miR‐21, 25.5 am for miR‐375, 59.8 am for miR‐10b, and 21.9 am for miR‐1246, surpassing conventional RCA in both LOD and linear dynamic range (Figures S7 and S8, Supporting Information). A comparative analysis with other Ago‐based, RCA‐based, and endonuclease‐based methods is summarized in Table S3 (Supporting Information). Furthermore, TACTIC demonstrated exceptional recognition specificity, enabling precise discrimination of single‐base mutations. Four kinds of miRNA sequences harboring different numbers of base mutations (M1–M4) (Figure 4E–H) and other miRNA analogs (Figure 4I–J) were used to challenge the specificity. Compared to conventional RCA, TACTIC exhibited a statistically significant reduction in fluorescence signal in response to single‐base mutations. This likely stems from reduced tolerance for base mismatches under elevated temperatures,^[^ ^36^ ^]^ further supporting that increasing the reaction temperature not only enhances detection sensitivity but also improves mismatch discrimination specificity.

To broaden the scope of detectable targets, KPC DNA and KRAS^G12D^ mRNA were selected as novel targets. As shown in Figure 4K, two gDNAs (G2/G3) were designed to be complementary to the KPC DNA. The TtAgo/G2 and TtAgo/G3 complex specifically recognized and cleaved the KPC DNA, generating a short ssDNA fragment (L1) bearing a 3′‐hydroxyl terminus. L1 was then hybridized with the circular template (synthesized by padlock‐KPC) to initiate the TACTIC assay. As shown in Figure 4L, fluorescence signal decreased gradually with diluted target DNA concentration. TACTIC achieved a LOD of 104.5 am for KPC DNA. As depicted in Figure 4M, a complementary gDNA (G4) was designed targeting the mutant mRNA. The TtAgo/G4 complex hybridized with and cleaved KRAS^G12D^ mRNA, releasing L2 to trigger TACTIC assay. Conversely, due to base mismatches at the cleavage site, the TtAgo/G4 complex failed to cleave wild‐type (WT) KRAS mRNA, thereby preventing subsequent signal output. Variant allele frequencies (VAFs) were set by mixing KRAS^G12D^ mRNA with WT KRAS mRNA and detected. As shown in Figure 4N, TACTIC successfully detected VAFs as low as 0.1%, while conventional RCA could only detect 5% VAFs. Finally, 40 clinical cases from different samples were collected, including 20 carbapenemase‐producing Klebsiella pneumoniae (CPKP) and 20 WT Klebsiella pneumoniae (KP), and genomic DNA was extracted. qPCR was first used to verify KPC gene expression in these samples (Figure 4O), followed by detection using the TACTIC assay. Meanwhile, the classic standard minimum inhibitory concentration (MIC) method was used to characterize these 40 samples. Detailed clinical data are presented in Table S4 (Supporting Information). As shown in Figure 4P–Q, receiver operating characteristic (ROC) curve results indicate that TACTIC distinguished KPC‐positive from KPC‐negative samples with 100% accuracy, which were fully consistent with those obtained by the MIC method. This consistency demonstrates the strong potential of TACTIC for the rapid and accurate analysis of CPKP.

### 2.6 BC Diagnosis in Cell Medias and Animal Models

**Figure 5:**
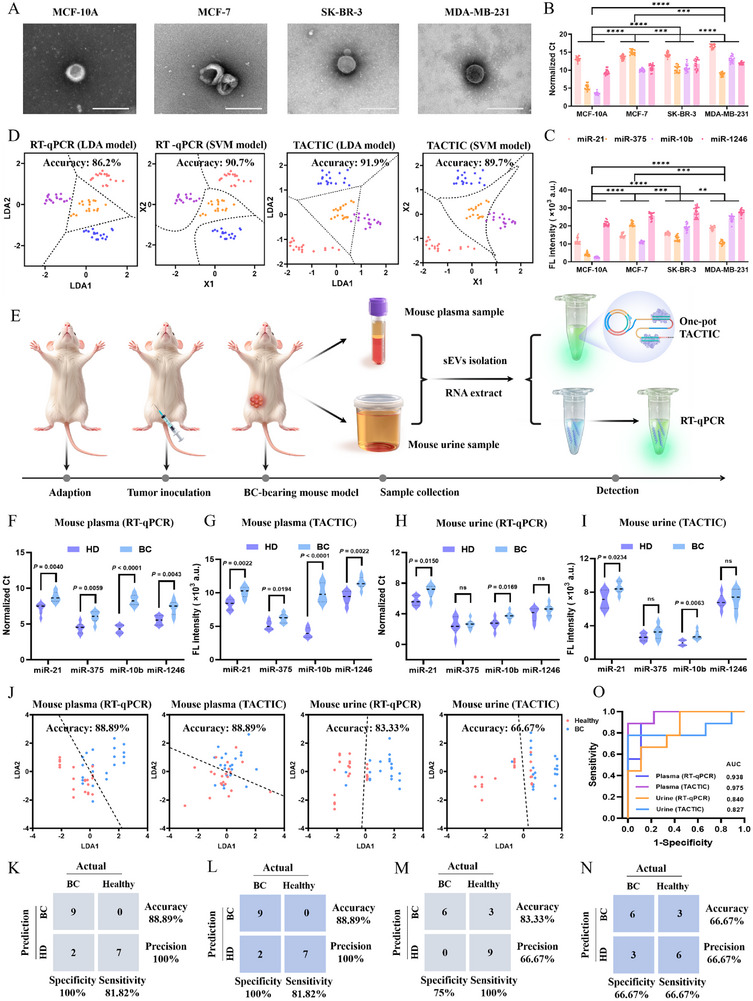
Application in BC Cells and BC‐bearing Mouse. A) TEM images of EVs derived from four different cell lines (MCF‐10A, MCF‐7, SK‐BR‐3, and MDA‐MB‐231). B) Expression profiles of the four EV miRNAs in the four cell lines validated by RT‐qPCR. The normalized Ct value was calculated by subtracting the acquired Ct value from the Ct value of 40. C) Expression profiles of the four EV miRNAs in the four cell lines validated by TACTIC. Bars represent the mean ± SD (*n* = 6). ^**^ *p* < 0.01, ^***^ *p* < 0.001, ^****^ *p* < 0.0001. D) Scatter plots from linear discriminant analysis (LDA) and support vector machine (SVM) based on RT‐qPCR (Ct) and TACTIC (fluorescence intensity) data. Dashed boundaries represent the decision planes for classifying the four cell types. Insets indicate model accuracy. E) Schematic diagram of establishing the BC‐bearing mouse model and collecting plasma and urine samples. F) Expression profiles of the four EV miRNAs in plasma samples from tumor‐bearing mice validated by RT‐qPCR. G) Expression profiles of the four EV miRNAs in plasma samples from tumor‐bearing mice validated by TACTIC. H) Expression profiles of the four EV miRNAs in urine samples from tumor‐bearing mice validated by RT‐qPCR. I) Expression profiles of the four EV miRNAs in urine samples from tumor‐bearing mice validated by TACTIC. J) Scatter plots from LDA based on RT‐qPCR (Ct) and TACTIC (fluorescence intensity) data. Dashed boundaries represent the decision planes for classifying healthy and BC‐bearing mice. Insets indicate model accuracy. K) Confusion matrix for the classification results of the dataset based on plasma EV miRNA detection by RT‐qPCR. L) Confusion matrix for the classification results of the dataset based on plasma EV miRNA detection by TACTIC. M) Confusion matrix for the classification results of the dataset based on urine EV miRNA detection by RT‐qPCR. N) Confusion matrix for the classification results of the dataset based on plasma EV miRNA detection by TACTIC. O) ROC analysis evaluating the diagnostic performance of RT‐qPCR and TACTIC in mouse plasma and urine samples.

TACTIC was applied to profile the expression of four EV miRNAs in three BC cell lines with distinct molecular phenotypes and one normal breast cell line. MCF‐7 (luminal), SK‐BR‐3 (HER2+), MDA‐MB‐231 (triple‐negative), and MCF‐10A were selected. EVs were collected from the four cell media via ultracentrifugation. TEM and NTA were employed to characterize the morphology and size of EVs derived from each cell line (**Figure** **5**A; Figure S9, Supporting Information). EVs surface marker proteins (TSG 101, CD 9, CD 81, CD 63) were further confirmed by western blotting (Figure S10, Supporting Information). Then, total miRNAs were extracted, and the distinct expression levels of the four miRNAs were validated using both RT‐qPCR and TACTIC, respectively. As shown in Figure 5B, C, the expression levels of all four EV miRNAs were higher in the BC cell lines compared to the MCF‐10A cells, with significant heterogeneity observed across the different BC cells. Subsequently, using the calibration curves established by RT‐qPCR (Figure S11, Supporting Information) and the linear regression equations derived from TACTIC, the concentration of the four EV miRNAs in the four cell lines was accurately quantified. Although there are slight systematic differences between the two methods with respect to certain indicators (Figure S12, Supporting Information), these discrepancies arise because TACTIC may impose more stringent requirements on miRNA molecular integrity‐particularly at the 3' end‐compared to RT‐qPCR. As a result, differences may occur between the “total” miRNAs detected by RT‐qPCR and the subset of functionally intact miRNAs captured by TACTIC. Overall, the results obtained using both methods demonstrate good consistency, further confirming the reliability of TACTIC.

To evaluate whether the expression patterns of the selected miRNA panels could distinguish BC cells with different molecular fingerprints (MCF‐7, SK‐BR‐3, MDA‐MB‐231) and discriminate BC cells from normal cells, we performed multiparametric analysis of the EVs miRNAs using linear discriminant analysis (LDA) and support vector machine (SVM). This integrated the expression signatures of the four EVs miRNAs to obtain optimal decision planes for classifying the four cell lines. As shown in Figure 5D, using the gold‐standard RT‐qPCR data, LDA and SVM achieved classification accuracies of 86.2% and 90.7%, respectively. Confusion matrices and ROC curve analyses confirmed that RT‐qPCR achieved 100% accuracy in distinguishing BC cell lines from normal cells (Figures S13 and S14, Supporting Information). Similarly, using TACTIC data, LDA and SVM yielded accuracies of 91.9% and 89.7%, respectively, results comparable to those obtained with RT‐qPCR. Confusion matrices and ROC analyses also confirmed that TACTIC achieved 100% accuracy in discriminating BC cell lines from healthy cells (Figures S15 and S16, Supporting Information). The slight differences in performance between the two classification methods may stem from their distinct modeling philosophies and optimization objectives. In summary, we demonstrated that multiplex miRNA analysis using TACTIC provides a reliable result for BC cell subtyping and classification.

In the field of liquid biopsy, plasma and urine represent two of the most important and increasingly utilized biofluid types. Urine, in particular, is emerging as a critical indicator for early disease diagnosis and prognosis assessment due to its non‐invasive nature and suitability for home collection.^[^ ^37^ ^]^ To validate the diagnostic potential of the TACTIC method, we established a BC‐bearing mouse model by orthotopically injecting MDA‐MB‐231 cells (Figure S17, Supporting Information). Plasma and urine samples were then collected from both tumor‐bearing mice (*n* = 6) and healthy mice (*n* = 6) (Figure 5E). We first characterized EVs isolated from both biofluid types using TEM and NTA (Figure S18, Supporting Information). Subsequently, the expression of the four EV miRNAs in both fluids was validated using both TACTIC and RT‐qPCR. As shown in Figure 5F–G, in mouse plasma samples, the expression profiles of all four EV miRNAs were significantly higher in BC‐bearing mice compared to healthy controls, with miR‐10b showing particularly pronounced upregulation. Results from both detection methods showed high consistency (Figure S19, Supporting Information). Published studies indicate that miR‐10b, acting as a key pro‐metastatic driver, is significantly associated with lymph node metastasis, distant metastasis, and reduced survival, and is markedly elevated in plasma EVs from metastatic breast cancer patients.^[^ ^38^ ^]^ In BC‐derived EVs, elevated expression of miR‐21 and miR‐1246 is highly prevalent and significant, and they are often detected in combination to enhance BC diagnostic efficacy.^[^ ^39^ ^]^ While the expression pattern of miR‐375 is unclear, literature reports show it is overexpressed in triple‐negative breast cancer.^[^ ^40^ ^]^ Therefore, the overexpression of these four EV miRNAs observed in the orthotopic xenograft model is consistent with results of these studies. Conversely, in mouse urine samples, the differences of EV miR‐21 and miR‐10b between cancerous mice and healthy mice were statistically significant, while the differences of EV miR‐375 and miR‐1246 were not statistically significant (Figure 5H,I). Furthermore, the concentrations of all four EV miRNAs in urine were significantly lower than those in plasma (Figure S20, Supporting Information). This phenomenon can be attributed to the extremely low concentration of EV miRNAs in urine, which is influenced by the renal filtration barrier and urine dilution. Additionally, urine‐based testing is mainly applicable to malignant tumors of the urinary system.^[^ ^41^ ^]^ Therefore, making a wise choice of sample type is crucial for BC analysis based on liquid biopsy.

Subsequently, the multidimensional expression profiles of the four EV miRNAs were projected into a 2D scatter plot using the first two linear discriminants (LD1 and LD2). LDA was employed to identify the decision plane that maximally discriminated between the healthy and BC groups (Figure 5J). The LDA‐derived decision planes and corresponding confusion matrix results demonstrated consistent accuracy (88.89%) in distinguishing BC groups from healthy groups in mouse plasma samples using both RT‐qPCR and TACTIC (Figure 5K,L). In contrast, for mouse urine samples, TACTIC achieved lower accuracy compared to RT‐qPCR (66.67% vs 83.33%) (Figure 5M,N). This can likely be attributed to the differing requirements for detecting target integrity between the two methods, as well as the subtle interference from potential inhibitors in the urine matrix on the coordinated activity of multiple enzymes within the TACTIC system. ROC analysis was further used to assess diagnostic performance. As shown in Figure 5O, TACTIC yielded AUC values of 0.975 and 0.824 in mouse plasma and urine samples, respectively, comparable to those obtained with RT‐qPCR. This result reaffirms the robust applicable capability of our method.

### 2.7 BC Diagnosis in Clinical Cohort

**Figure 6:**
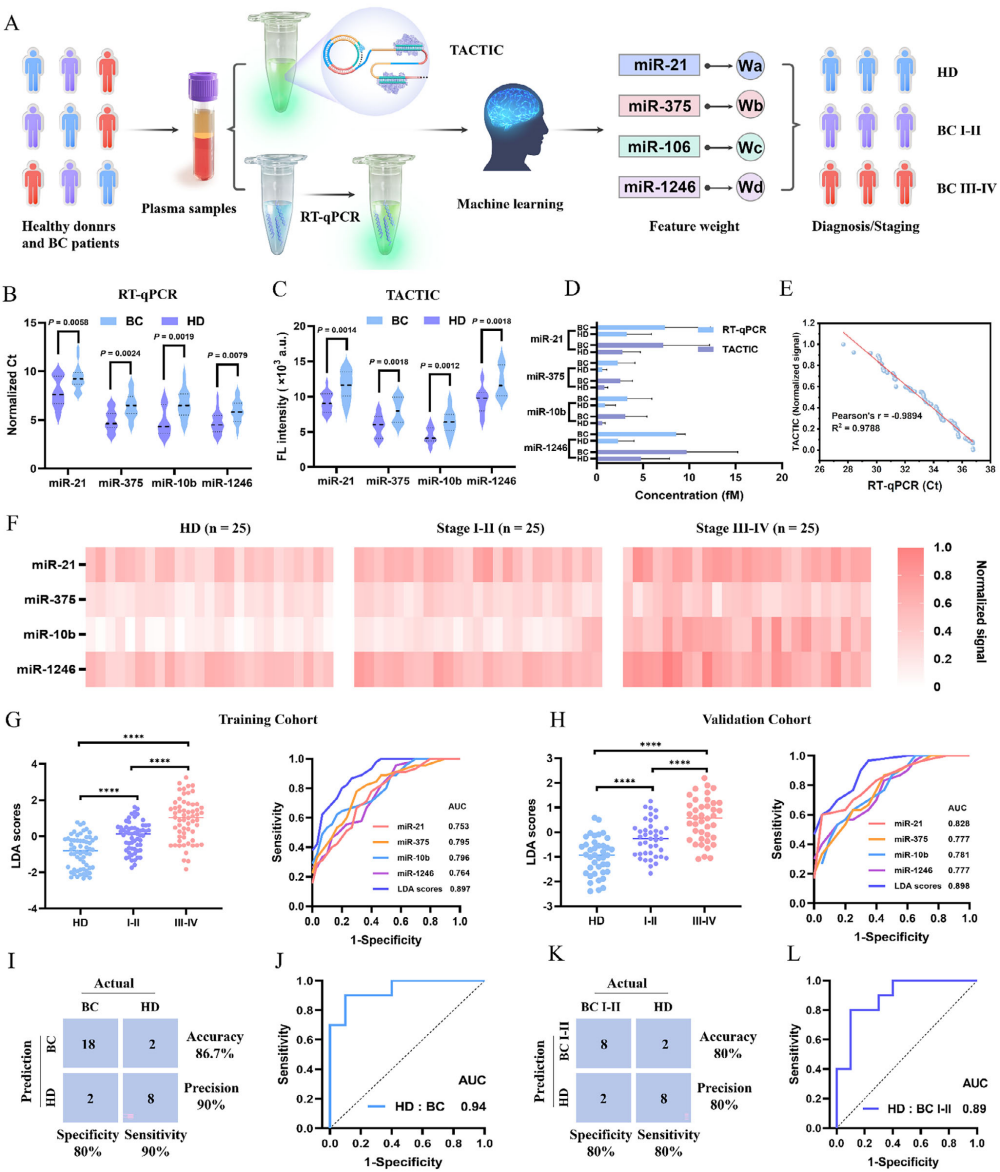
Application in the clinical cohort. A) Schematic diagram of clinical plasma sample collection, detection, and machine learning model establishment. B) Expression profiles of the four EV miRNAs in HD (*n* = 15) and BC patients (*n* = 15) validated by RT‐qPCR. C) Expression profiles of the four EV miRNAs in HD and BC patients validated by TACTIC. D) Consistency analysis of the concentrations of the four EV miRNAs in HD and BC patients detected by RT‐qPCR and TACTIC. E) Linear regression analysis between the two detection methods. F) Expression profiles of the four EV miRNAs in the clinical cohort (*n* = 75) analyzed by TACTIC. G) LDA scores in the training cohort for discriminating HD (*n* = 15), stage I, II BC patients (*n* = 15), and stage III, IV BC patients (*n* = 15). ROC analysis of the performance of individual miRNAs and the LDA score in discriminating HD, stage I, II BC, and stage III, IV BC patients within the training cohort. H) LDA scores in the validation cohort for discriminating HD (*n* = 10), stage I, II BC patients (*n* = 10), and stage III, IV BC patients (*n* = 10). ROC analysis of the performance of individual miRNAs and the LDA score in discriminating HD, stage I, II BC, and stage III, IV BC patients within the validation cohort. I) Confusion matrix for the classification results of the dataset based on the detection of clinical plasma EV miRNAs from BC patients using TACTIC. J) ROC analysis of the performance of the LDA score in discriminating HD from BC patients in the validation cohort. K) Confusion matrix for the classification results of the dataset based on the detection of clinical plasma EV miRNAs from early‐stage BC patients using TACTIC. L) ROC analysis of the performance of the LDA score in discriminating HD from early‐stage BC patients in the validation cohort.

To evaluate the clinical diagnostic potential of the TACTIC method, plasma samples were collected from healthy donors (HD) and BC patients (**Figure** **6**A). We initially analyzed plasma samples from 15 HD and 15 BC patients by TACTIC and RT‐qPCR. As shown in Figure 6B,C, both fluorescence signals and normalized Ct values were significantly higher in BC patients compared to HD. The high consistency between the two methods demonstrated the reliability of TACTIC for clinical diagnostics (Figure 6D,E). Subsequently, a clinical cohort consisting of 75 individuals was established: 25 HD, 25 stage I‐II BC patients, and 25 stage III‐IV BC patients. The expression profiles of the four EV miRNAs in all 75 plasma samples were quantified using normalized fluorescence signals, and significant heterogeneity was found among different cancer stages (Figure 6F; Figure S21, Supporting Information). ROC analysis was employed to assess the diagnostic and early detection capabilities of each individual miRNA biomarker. Results indicated that no single biomarker demonstrated satisfactory diagnostic performance (Figure S22, Supporting Information). Therefore, we utilized machine learning for multiparametric analysis to enhance diagnostic accuracy.

We first applied LDA to classify using a training cohort of 45 samples (15 HD, 15 stage I‐II BC, and 15 stage III‐IV BC). As depicted in Figure 6G, the LDA scores demonstrated high performance (84.4% accuracy, 90% sensitivity, 73.3% specificity), effectively distinguishing stage I, II BC, stage III, IV BC patients, and HD (Figure S23A, Supporting Information). Notably, the LDA score achieved an AUC value of 0.897, significantly surpassing the performance of any single miRNA biomarker. The LDA model was then applied to an independent validation cohort (10 HD, 10 stage I, II BC, and 10 stage III, IV BC). As shown in Figure 6H, the LDA scores effectively differentiated stage I, II BC, stage III, IV BC patients, and HD, achieving 80% accuracy, 80% sensitivity, and 80% specificity (Figure S23B, Supporting Information). ROC analysis yielded an AUC value of 0.898. To specifically evaluate the method's ability to diagnose BC patients and detect early‐stage BC, data from the validation cohort were further analyzed (Figure 6I–L). Confusion matrix and ROC results revealed that TACTIC achieved 86.7% accuracy, 90% sensitivity, and 80% specificity for distinguishing BC patients from HD (AUC = 0.94), and 80% accuracy, 80% sensitivity, and 80% specificity for detecting early‐stage (stage I, II) BC patients (AUC = 0.89). In summary, these results further demonstrate the applicability of the TACTIC method for the clinical diagnosis of EV miRNAs and its potential to enhance early‐stage cancer detection performance.

## 3 Discussion

In conclusion, this study successfully established a novel integrated one‐pot isothermal platform with thermal‐enhanced kinetics for ultrasensitive and specific nucleic acid detection. By leveraging the synergistic interaction between the thermophilic TtAgo and RCA, we effectively transformed conventional linear amplification into an exponential process. The thermal stability of TtAgo enables the reaction to be conducted at 70 °C, a condition that plays a critical role in disrupting secondary structures within the circular template, suppressing non‐specific amplification, and substantially improving amplification efficiency. Simultaneously, TtAgo's programmable endonuclease activity continuously cleaved RCA products, generating abundant secondary primers that fueled positive feedback amplification. This dynamic thermal‐mediated mechanism operates within a single tube under precisely controlled kinetic equilibrium, thereby ensuring robust analytical performance.

TACTIC demonstrated remarkable analytical capabilities, achieving attomolar (am) sensitivity and single‐base discrimination specificity across diverse nucleic acid targets. With a streamlined 30 min workflow, it eliminates the need for manual intervention and minimizes the risk of cross‐contamination associated with multi‐step procedures, offering significant practical advantages. Importantly, the robustness of the platform has been validated in complex biological matrices, including cell lines, in situ mouse models (plasma and urine‐derived extracellular vesicles), and clinical patient cohorts. Quantitative results for target EV miRNA expression obtained using this platform show high concordance with those from the gold‐standard RT‐qPCR method. Furthermore, integration with machine learning‐based multi‐parameter analysis enhances its diagnostic potential. An LDA model trained on multiple EV miRNA features achieves high accuracy, sensitivity, and specificity in distinguishing BC patients from HD and in detecting early‐stage disease. This platform establishes a reliable and versatile foundation for point‐of‐care testing, biomarker validation, and precision oncology, particularly in the context of early cancer detection and molecular subtyping based on liquid biopsy markers.

## 4 Experimental Section

### Materials

All synthetic DNA and RNA oligonucleotides were purchased from Sangon Biotech (Shanghai, China). Detailed nucleotide sequences are provided in Tables S1 and S2 (Supporting Information). T4 DNA ligase, Exonuclease I/Exonuclease III, SplintR Ligase, TtAgo, Bst 3.0 DNA polymerase, nicking endonucleases (Nb.BsrDI, Nb.BtsI, and Nb.BsmI), and their supplied buffers were obtained from New England Biolabs (NEB, USA). SYBR Green I dye (20×) was purchased from G‐clone (Beijing, China). The LV Exosome Isolation Kit V1, WisExo Exosome Isolation Kit (Precipitation), and Universal RNA Extraction Kit were acquired from WisDelivery Biotech (Wuhan, China). The Monarch Plasmid Miniprep Kit was ordered from New England Biolabs (NEB, USA). The miRNA First Strand cDNA Synthesis Kit (Stem‐loop Method) was sourced from Sangon Biotech (Shanghai, China). The TB Green Premix Ex TaqII Kit was purchased from Takara Biotech (Dalian, China). The BALB/c nude mice (*n* = 12, female, 4–6 weeks) were bought from Cavens (Changzhou, China).

### Circular Template Preparation

The linear single‐stranded padlock probe was cyclized with the assistance of a DNA primer by using a classical denaturation‐annealing protocol. Briefly, 1 µL padlock probe (20 µM) was mixed with an equimolar amount of DNA bridge primer (BP) and supplemented with nuclease‐free water to a final volume of 12 µL. The mixture was incubated at 95 °C for 5 min and then rapidly cooled to room temperature. Subsequently, 1 µL of T4 DNA ligase (400 U) and 2 µL of ligase buffer were added. After thorough mixing, the reaction was incubated overnight (12 h) at 16 °C, followed by heat inactivation at 65 °C for 20 min. Finally, 2 µL of Exonuclease I (40U), 1 µL of Exonuclease III (100 U), and 2 µL of Exonuclease I buffer were added to the mixture and incubated overnight (12 h). The exonucleases were then inactivated by heating at 80 °C for 20 min. The resulting circular template (1 µM) was stored at −20 °C until use.

### TACTIC Assay and Multi‐Step Protocol Workflows

One‐Step TACTIC Assay: A 20 µL TACTIC reaction mixture was prepared on ice, containing: 2 µL TtAgo protein (1 M), 1 µL G1 (1 M), 2 µL Thermopol buffer (10×), 2 µL circular template (1 µM), 2 µL dNTP (1 mm), 2 µL RP (1 µM), 0.5 µL of Bst 3.0 DNA polymerase (4 U), 1 µL of SYBR Green I dye (20×), 1 µL of target miRNA at varying concentrations, supplemented nuclease‐free water to a final volume of 20 µL. After mixing, the sample was immediately transferred to a Gene‐8C isothermal fluorescence detector (Hangzhou, China) and incubated at 70 °C. Real‐time fluorescence intensity was monitored every 15 s. Endpoint fluorescence spectra were obtained using a F‐7000 fluorescence spectrophotometer (Excitation: 425 nm, Emission: 450–600 nm). Two‐Step Assay: Target‐induced padlock probe ligation and the subsequent RCA/TtAgo reaction were performed sequentially. Ligation Step: 1 µL of target miRNA and 1 µL of padlock 1–9 (1 µM) were denatured at 95 °C for 5 min and then annealed to room temperature. The mixture was transferred to a 10 µL ligation reaction containing 1 µL of SplintR Ligase (25 U) and 1 µL of SplintR Ligase buffer. The reaction proceeded at 37 °C for 2 h and then 65 °C for 20 min, yielding the RNA‐ligated circular template. Detection Step: The entire 10 µL ligation mixture was added to the detection mixture containing 2 µL TtAgo, 0.5 µL G1 (2 µM), 2 µL of Thermopol buffer, 2 µL of dNTP, 2 µL RP, 0.5 µL of Bst 3.0 DNA polymerase, 1 µL of SYBR Green I dye. Real‐time fluorescence intensity was monitored identically to the one‐step assay protocol. Three‐Step Assay: Target‐induced padlock probe ligation, RCA, and TtAgo cleavage were performed in separate steps. Ligation Step: Identical to Step 1 of the Two‐Step assay, yielding the RNA‐ligated circular template (10 µL). RCA Step: The entire ligation mixture was added to an RCA mixture containing 2 µL of Thermopol buffer, 2 µL of dNTP, 0.5 µL of Bst 3.0 DNA polymerase. The mixture was incubated at 37 °C for 1 h and then 80 °C for 5 min. Detection Step: 2 µL of TtAgo, 0.5 µL of G1, and 2 µL of RP were added to the 14.5 µL RCA product mixture. Real‐time fluorescence intensity was monitored identically to the one‐step assay protocol.

### Native Gel Electrophoresis

12% PAGE gel was performed to verify the feasibility of the experiment. 9 µL of the reaction sample was mixed with 1 µL of 10× loading buffer and analyzed in 1 × TBE buffer for 45 min (110 V). The gel was then stained using GelRed nucleic acid staining. Gel imaging was conducted on a Bio‐Rad ChemDoc XRS imaging system.

### Cell Culture and EVs Isolation

MCF‐7, SK‐BR‐3, and MDA‐MB‐231 cells were cultured in complete DMEM medium supplemented with 10% fetal bovine serum (FBS) and 1% penicillin‐streptomycin. MCF‐10A cells were cultured in specialized MCF‐10A growth medium. All cell lines were maintained in a humidified incubator at 37 °C with 5% CO_2_. When cells reached ≈70% confluence, they were washed and incubated in serum‐free medium for 48 h prior to EVs collection. Cell culture supernatants were then collected and subjected to sequential centrifugation: 5 min at 300 g followed by 10 min at 3,000 g to remove cells and cellular debris. All the centrifugations were performed at 4°C. The clarified supernatant was filtered through a 0.45 µm filter (BioSharp, China). EVs were subsequently isolated from the filtered supernatant using an EV Isolation Kit (anion‐exchange chromatography) according to the protocol. Isolated EVs were stored at −80 °C for subsequent analysis. Mouse plasma, urine, and clinical plasma samples were processed by sequential centrifugation: 5 min at 300 g followed by 10 min at 2,000 g to remove cells and debris. Large vesicles were then pelleted by centrifugation at 14, 000 g for 30 min. EVs were finally isolated from the resulting supernatant using an EV Isolation Kit (precipitation‐based method) following the manufacturer's protocol.

Characterization of EVs: EVs derived from cell culture medium, mouse plasma, and urine were negatively stained and characterized for morphology by transmission electron microscopy (TEM, FEI Tecnai G2 12). Nanoparticle tracking analysis (NTA, ZetaView PMX 110) was employed to analyze the particle concentration and size distribution of EVs. Ultimately, Western blotting (WB) was used to detect the expression of characteristic EV surface protein markers.

### miRNA Extraction and RT‐qPCR

EV miRNAs were purified from 100 µL of isolated EV solution using a universal RNA extraction kit and eluted in 30 µL of nuclease‐free water according to the protocol. miRNA extracts were then reverse transcribed into cDNA using the miRNA First Strand cDNA Synthesis Kit (Stem‐loop Method) under the following conditions: 16 °C for 30 min, 37 °C for 30 min, and 85 °C for 5 min. qPCR was performed on the resulting cDNA using the TB Green Premix Ex TaqII Kit on a CFX‐96 Real‐Time System (Bio‐Rad). The thermal cycling protocol consisted of an initial denaturation step at 95 °C for 30 s, followed by 40 cycles of denaturation (95 °C for 5 s) and combined annealing/extension (60 °C for 30 s).

### Extraction of Bacterial Genomic DNA and qPCR

Forty clinical samples (including urine, blood, wound exudates, sputum, and pus) underwent bacterial culture and identification. Detailed information is provided in Table S4 (Supporting Information). This study was approved by the Ethics Committee of the First Affiliated Hospital of Army Medical University. Bacterial strains identified as carbapenemase‐producing Klebsiella pneumoniae (CPKP) and WT Klebsiella pneumoniae (KP) were cultured on blood agar plates until reaching the mid‐logarithmic growth phase. Colonies selected from each culture plate were collected, resuspended in 0.9% NaCl buffer, and adjusted to a concentration of 3 × 10 CFU/mL using McFarland turbidity standards. Genomic DNA was extracted following the protocol of the Monarch Plasmid Miniprep Kit, with DNA concentration quantified using a NanoDrop spectrophotometer (Thermo Fisher Scientific, USA). Gene‐specific primers were subsequently designed for qPCR to validate the expression of the KPC gene. The detection protocol employed identical qPCR conditions as described above.

### Establishment of the BC‐Bearing Mouse Model

All animal experiments were approved by the Institutional Animal Welfare Ethics Committee and the Ethics Committee of the Army Medical University, and were conducted in accordance with the Guide for the Care and Use of Laboratory Animals (Approval Number: AMUWEC20225084). Twelve 4‐week‐old female nude mice were randomly assigned to either a tumor‐bearing group or a healthy control group. After a one‐week acclimatization period, mice in the tumor‐bearing group were orthotopically injected with MDA‐MB‐231 cells (4.5 × 10^6^ cells). Tumor volume was monitored and measured weekly. Tumor volume was calculated using the formula: (a × b^2^)/2, where a represents the long axis and b represents the short axis. Once the tumor volume reached 1000 mm^3^, urine samples were collected from mice in both groups using metabolic cages (Yuyan, Shanghai). Finally, blood plasma was collected from all mice via cardiac puncture, and the mice were subsequently sacrificed.

### Clinical Sample Collection

All clinical plasma samples (*n* = 105) used in this study were obtained from the First Affiliated Hospital of Army Medical University. The study protocol was formally approved by the Ethics Committee of the First Affiliated Hospital of Army Medical University (Approval No.: KY2025140). The clinical samples were divided into two cohorts. Cohort 1: Comprised HD (*n* = 15) and BC patients (*n* = 15). Samples from this cohort were analyzed by both TACTIC and RT‐qPCR to validate method concordance. Cohort 2: Comprised HD (*n* = 25), BC patients in stage I, II (*n* = 25), and BC patients in stage III, IV (*n* = 25). Machine learning‐driven diagnostic and staging models for BC were developed and evaluated using a training set (15 HD, 15 in stages I, II, 15 in stages III, IV) and an independent validation set (10 HD, 10 in stages I, II, 10 in stages III, IV) derived from Cohort 2. Detailed clinical information is provided in Table S5 (Supporting Information).

### Machine Learning Modeling

For cell media and mouse plasma/urine sample analysis, the original data underwent Gaussian noise augmentation while preserving the original class distribution. Data augmentation was performed only on the training set. The augmented dataset was then partitioned into training and validation sets at a 7:3 ratio. Linear Discriminant Analysis (LDA) and Support Vector Machine (SVM) were employed to identify optimal decision planes that maximally discriminate each class from the others. For the clinical cohort analysis, the dataset was partitioned into training and validation sets at a 7:3 ratio. Z‐score normalization was applied separately to the training and validation sets. Model parameters were optimized using 3‐fold cross‐validation. Considering the LDA model's superior stability, strong interpretability, and advantages in visualization and deployment‐particularly in small‐to medium‐sized clinical datasets‐it is selected as the core algorithm for systematic evaluation of its performance in breast cancer diagnosis and staging within both the training and independent validation cohorts. Initial LDA models were built based on individual features, with parameter optimization performed for each model. Finally, a combined model utilizing LDA scores was constructed based on the top four individual‐feature models.

### Statistical Analysis

Data are presented as the mean ± standard deviation (s.d.) from three independent replicates. All statistical analyses were performed with a 95% confidence interval (*p* < 0.05) using GraphPad Prism (version 9.5.1) and SPSS 27 software. Significance between groups was assessed using Student's *t*‐test or two‐way analysis of variance (ANOVA).

## Conflict of Interest

The authors declare no conflict of interest.

## Supporting information



Supporting Information

## Data Availability

The data that support the findings of this study are available from the corresponding author upon reasonable request.
