# Supplementary material for: A TtAgo‐Driven Autocatalytic Circuit with Thermal‐Enhanced Kinetics for One‐Pot Nucleic Acid Detection
Source: Adv Sci (Weinh). 2025 Dec 12;13(9):e21671. doi: 10.1002/advs.202521671 (PMC12904063; doi:10.1002/advs.202521671)
Supplement: Supplementary file 1 — Supporting Information [file ADVS-13-e21671-s001.pdf]

Supporting Information

A TtAgo-Driven Autocatalytic Circuit with Thermal-enhanced kinetics for One-pot Nucleic Acid Detection

Zuowei Xie, Ruijia Deng, Ben Niu, Yingjie Yang, Shuang Zhao, Hongzhao Yang, Meilin Gong, Jie Luo, Yu Tang, Jing Sheng, Yan Pi*, Ming Chen*, Kai Chang*

Z. Xie, R. Deng, B. Niu, Y. Yang, S. Zhao, H. Yang, M. Gong, J. Luo, Y. Tang, J. Sheng, M. Chen, K. Chang

Department of Clinical Laboratory Medicine, Southwest Hospital, Third Military Medical University (Army Medical University), Chongqing 400038, China

Email: chenming1971@tmmu.edu.cn; changkai0203@tmmu.edu.cn

Y. Pi

Department of Rehabilitation Medicine, The First Affiliated Hospital of Chongqing Medical University, Chongqing 400042, China

Email: cqmuyan@hospital.cqmu.edu.cn

K. Chang

State Key Laboratory of Trauma and Chemical Poisoning, Army Medical University, 30 Gaotanyan, Shapingba District, Chongqing 400038, China

Table of Contents

Figure S1. Fluorescence spectra in RCA and TACTIC assay.................................. S-3

Figure S2. Characterization of the circular template.. S-4

Figure S3. Probe structure prediction... S-5

Figure S4. Stability analysis of circular templates..S-6

Figure S5. Exploration of TtAgo cleavage sites... S-7

Figure S6. Optimization of experimental conditions..S-8

Figure S7. Evaluation of RCA detection performance... S-9

Figure S8. Comparison of detection limits.. S-10

Figure S9. NTA analysis of EVs derived from different cell sources.................... S-11

Figure S10. Western blotting analysis of specific EVs protein expression.................... S-12

Figure S11. Ribbon representation of the simulated structure at different times.... S-13

Figure S12. Heat map analysis..S-14

Figure S13. Identification of BC cell lines using RT-qPCR..................................S-15

Figure S14. Identification of BC cells with different molecular fingerprints..........S-16

Figure S15. Identification of BC cell lines using TACTIC.................................... S-17

Figure S16. Identification of BC cells with different molecular fingerprints..........S-18

Figure S17. BC-bearing mouse model..S-19

Figure S18. Characterization of EVs from plasma and urine of BC-bearing mice. S-20

Figure S19. Consistency analysis in mouse plasma samples....................................S-21

Figure S20. Consistency analysis in mouse urine sample....................................... S-22

Figure S21. The expression profiles of four EV miRNAs in different BC stage.... S-23

Figure S22. ROC analysis using single marker in 75 clinical cohort...................... S-24

Figure S23. Confusion matrix results in differentiating BC patients from HD....... S-25

Table S1. The sequences used in this work..S-26

Table S2. The sequences used for RT-PCR.. S-30

Table S3. Comparison with other methods.. S-31

Table S4. Clinical bacteria sample information.. S-32

Table S5. Clinical plasma sample information... S-34

Reference. S-40

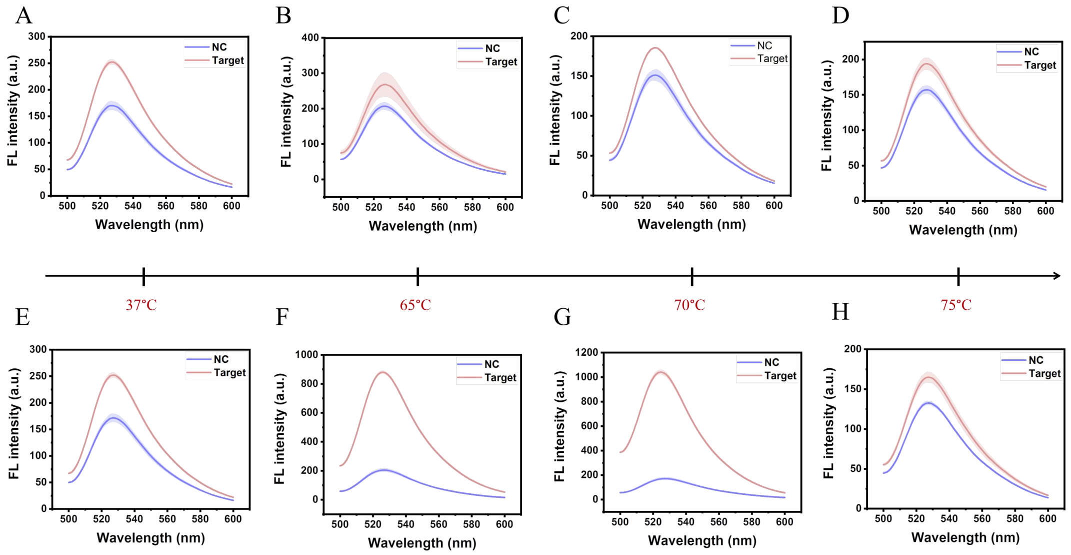

Figure S1. Fluorescence spectra in RCA and TACTIC assay. (A-D) Fluorescence spectra of conventional RCA under different reaction temperature conditions; (E-H) Fluorescence spectra of TACTIC under different reaction temperature conditions. The temperature gradient was set to 37°C, 65°C, 70°C, and 75°C. The concentrations of miR-21, padlock 1, TtAgo, G1, RP, and Bst 3.0 DNA polymerase were 1 nM, 100 nM, 100 nM, 25 nM, 100 nM, and 0.1 U/μL, respectively. Bars represent the mean ± SD (n = 3).

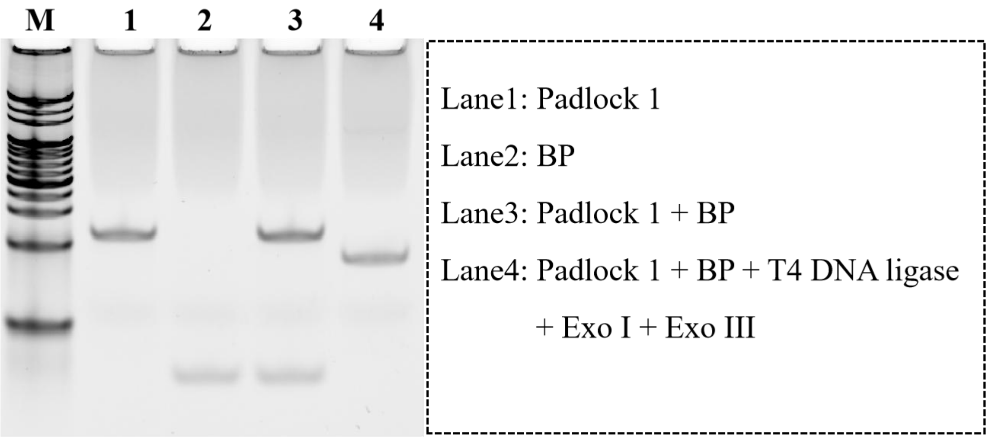

Figure S2. 12% PAGE characterization of the process of synthesizing the linear padlock probe into a circular template. The concentrations of padlock 1, BP, T4 DNA ligase, Exo I, and Exo III were 200 nM, 200 nM, 400 U/μL, 40 U/μL, and 100 U/μL, respectively.

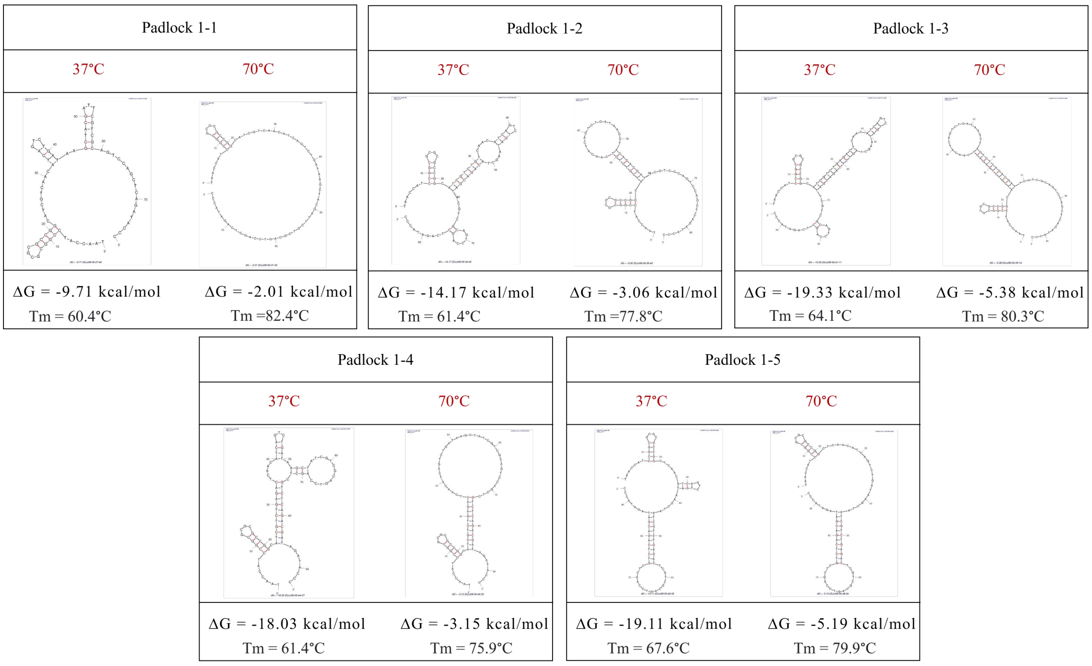

Figure S3. Probe structure prediction. Mfold (https://www.unafold.org/) was used to predict the free energy and Tm of five redesigned padlock probes at 37°C and 70°C.

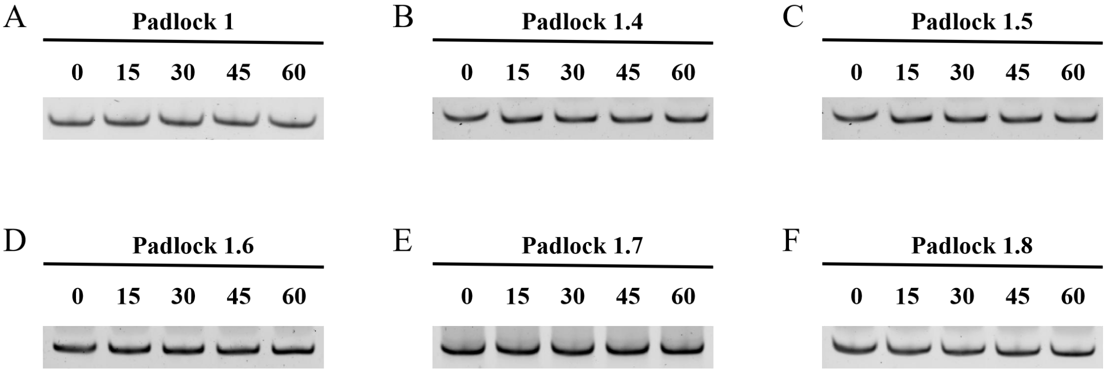

Figure S4. PAGE analysis of time-dependent behavior of circular templates (synthesized by different padlock probes). The time intervals were set at 0, 15, 30, 45, and 60 minutes, respectively. The concentrations of circular templates were kept at 200 nM.

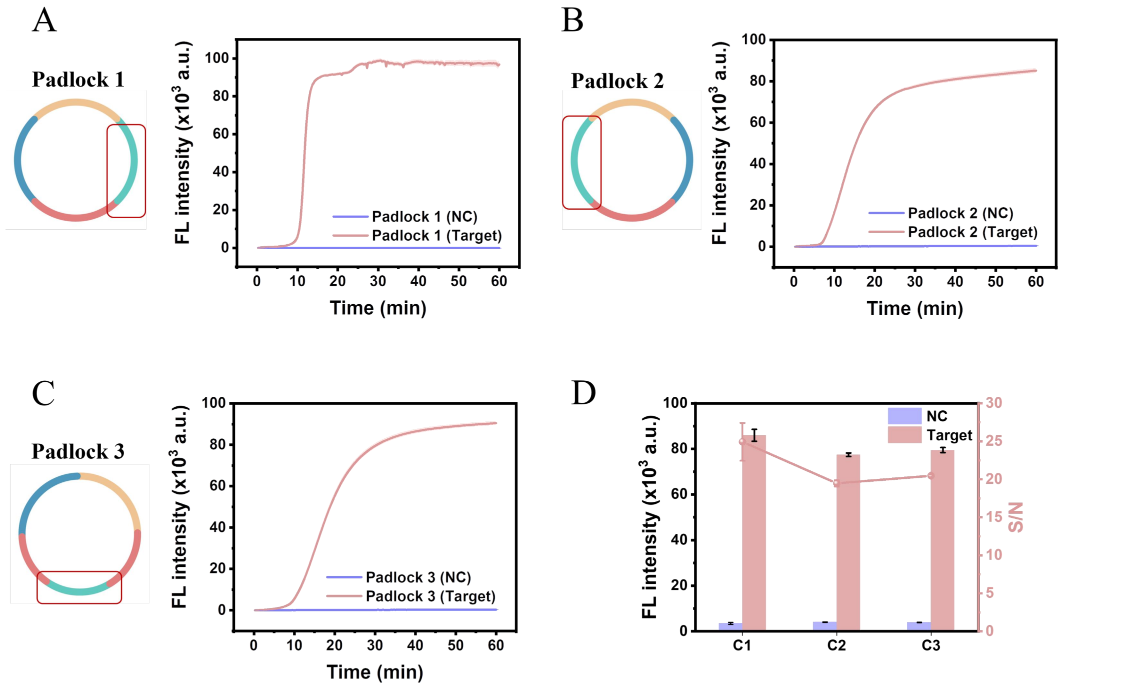

Figure S5. Exploration of TtAgo cleavage sites. Three different padlock probes were designed to evaluate the effect of TtAgo cleavage sites on amplification efficiency. (A) Right side; (B) Left side; (C) Middle; (D) Comparison of the signal-to-noise ratio based on fluorescence intensity of the three probes at 30 minutes. The concentrations of miR-21, padlock probes, TtAgo, gDNA1, RP, and Bst 3.0 DNA polymerase were 1 nM, 100 nM, 100 nM, 25 nM, 100 nM, and 0.2 U/μL, respectively. Bars represent the mean ± SD (n = 3).

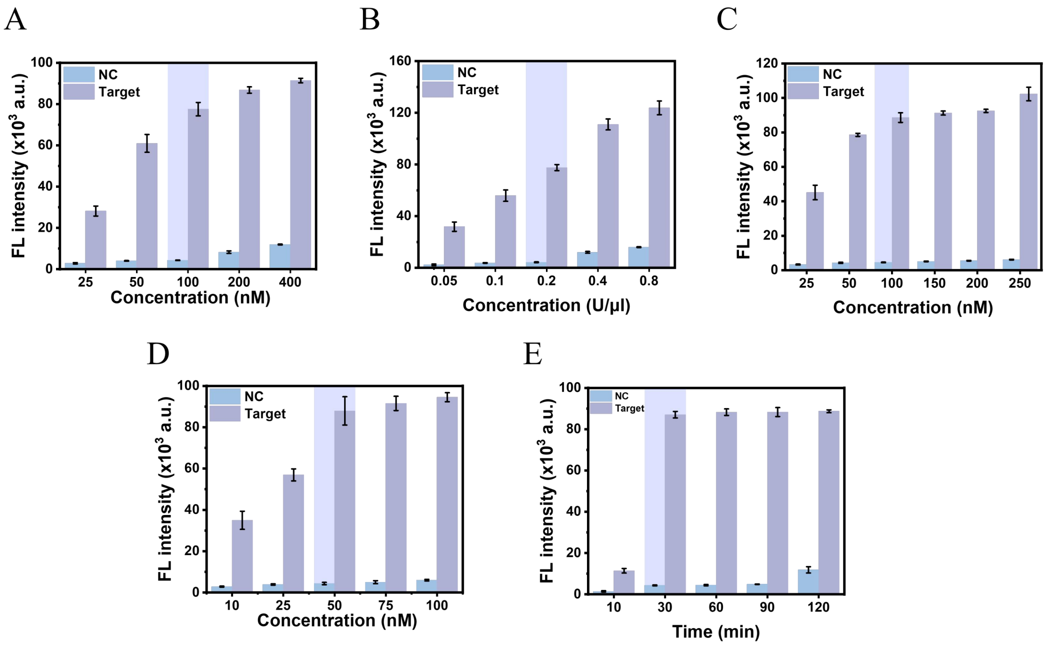

Figure S6. Optimization of experimental conditions. (A) Optimization of padlock 1 concentration; (B) Optimization of Bst 3.0 DNA polymerase concentration; (C) Optimization of TtAgo concentration; (D) Optimization of G1 concentration; (E) Optimization of reaction time. Bars represent the mean ± SD (n = 3).

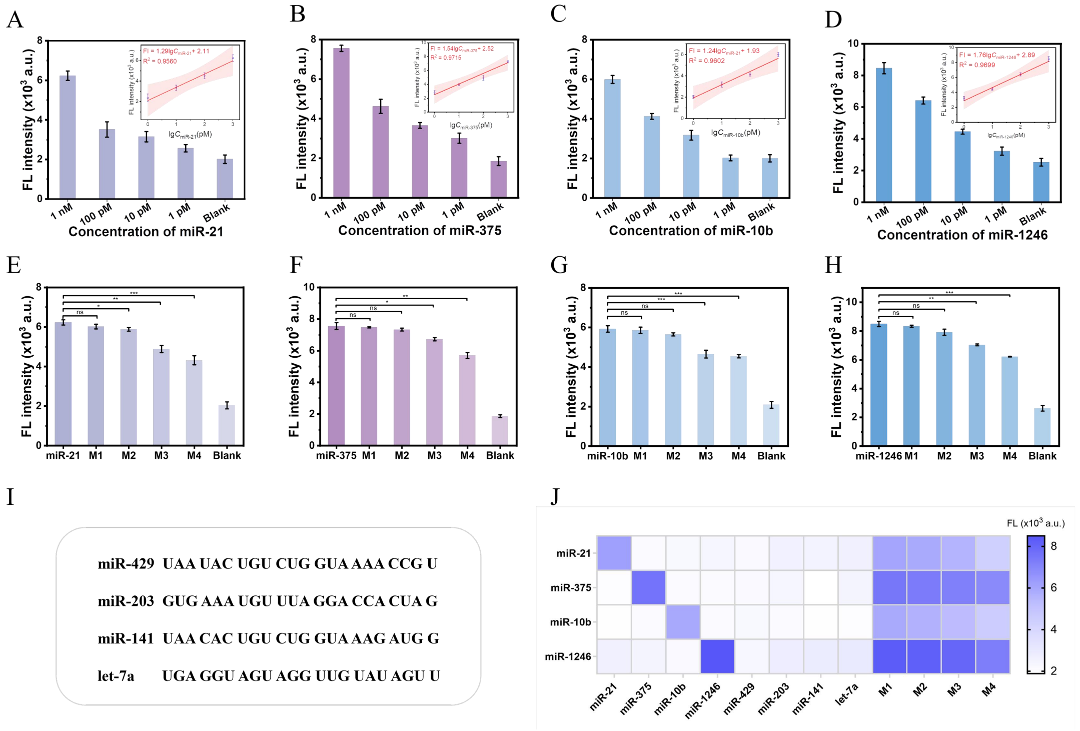

Figure S7. Evaluation of RCA detection performance. (A-D) Sensitivity analysis targeting miR-21, miR-375, miR-10b, and miR-1246, respectively; (E-H) Analysis of base mutation discrimination ability targeting miR-21, miR-375, miR-10b, and miR-1246, respectively. (I) Detailed base sequences of miRNA homologs; (J) RCA specificity heatmap. The concentration gradients of the four miRNAs were set at 1 nM, 100 pM, 10 pM, and 1 pM. M1 – M4 represent different numbers of base mutations. The concentrations of target miRNA and other miRNA homologs were maintained at 1 nM and 10 nM, respectively.

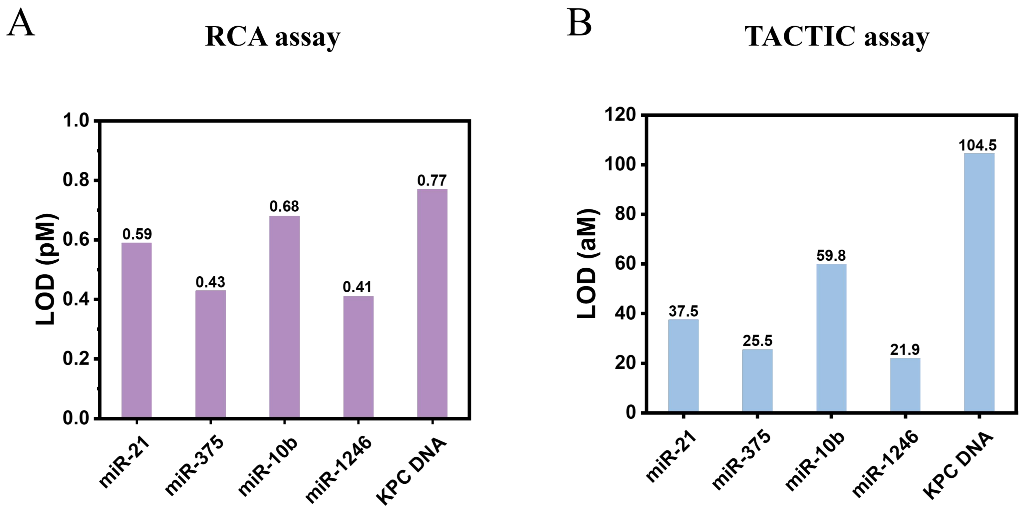

Figure S8. Comparison of detection limits. (A) Detection limit of the RCA assay; (B) Detection limit of the TACTIC assay.

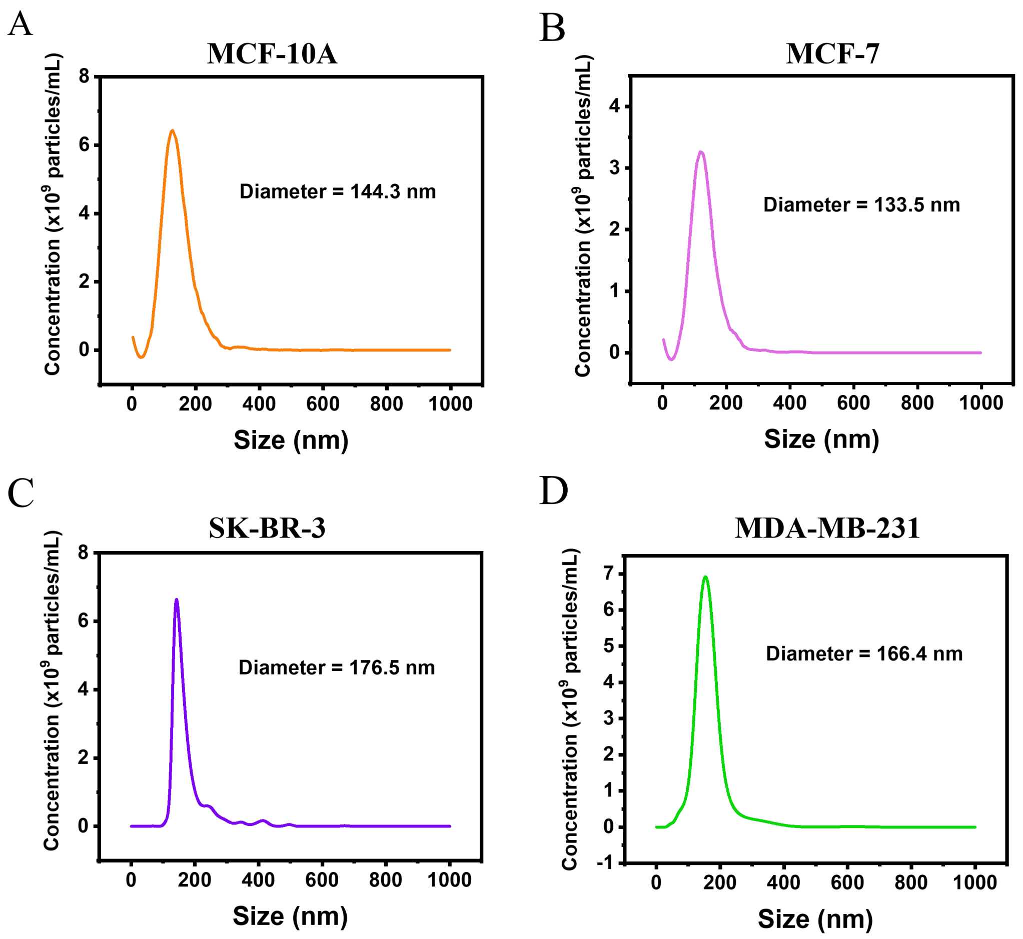

Figure S9. NTA analysis of EVs derived from different cell sources. (A) EVs derived from MCF-10A; (B) EVs derived from MCF-7; (C) EVs derived from SK-BR-3; (D) EVs derived from MDA-MB-231.

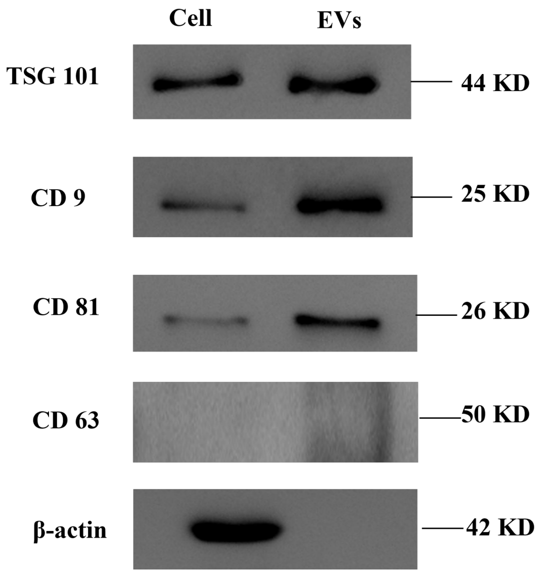

Figure S10. Western blotting analysis of specific EVs protein expression.

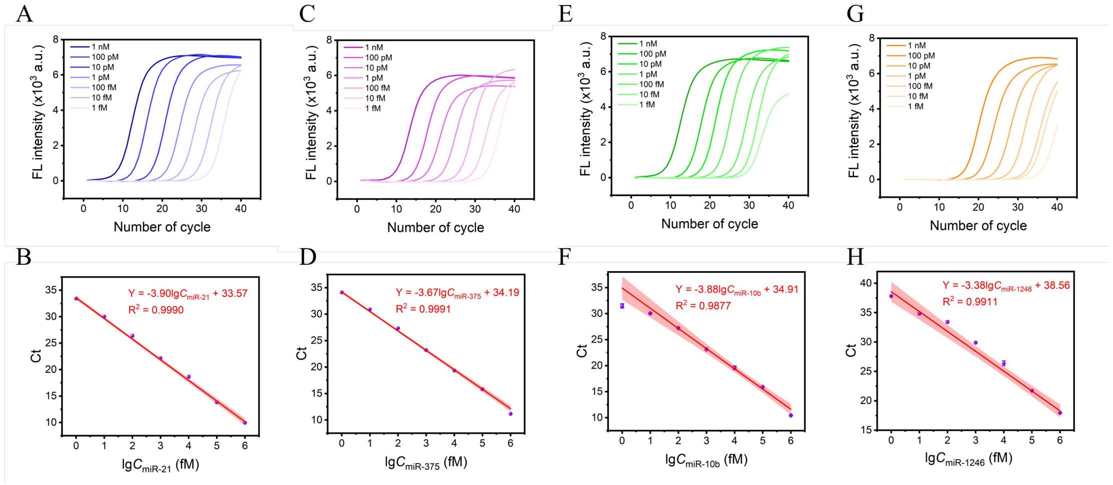

Figure S11. Establishment of RT-qPCR standard curves. (A-B) Real-time fluorescence and corresponding linear regression equation for different concentrations of miR-21; (C-D) Real-time fluorescence and corresponding linear regression equation for different concentrations of miR-375; (E-F) Real-time fluorescence and corresponding linear regression equation for different concentrations of miR-10b; (G-H) Real-time fluorescence and corresponding linear regression equation for different concentrations of miR-1246.

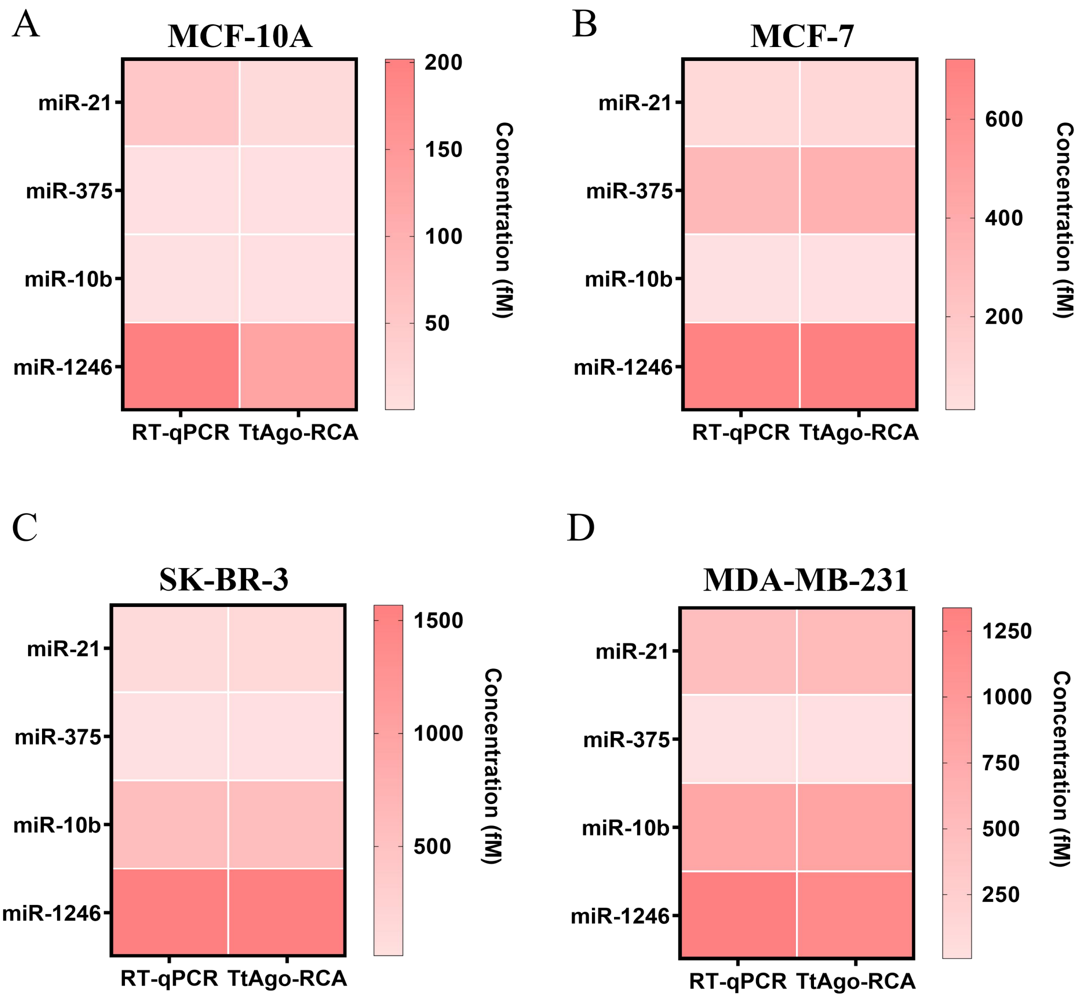

Figure S12. Heat map analysis. (A) Heat map of quantifying four miRNAs concentration by RT-qPCR and TACTIC in MCF-10A. (B) Heat map of quantifying four miRNAs concentration by RT-qPCR and TACTIC in MCF-7. (C) Heat map of quantifying four miRNAs concentration by RT-qPCR and TACTIC in SK-BR-3. (D) Heat map of quantifying four miRNAs concentration by RT-qPCR and TACTIC in MDA-MB-231.

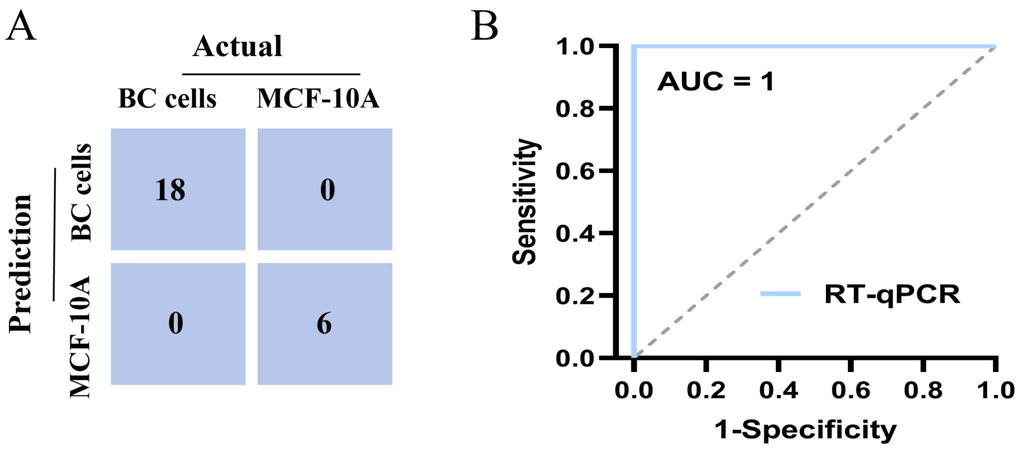

Figure S13. Identification of BC cell lines using RT-qPCR. (A) Confusion matrix results and (B) ROC analysis.

RT-qPCR

A

LDA	Actual: MDA-MB-231	Actual: MCF-7	Actual: SK-BR-3	Actual: MCF-10A
Prediction: MDA-MB-231	21	1	3	0
Prediction: MCF-7	1	21	4	0
Prediction: SK-BR-3	1	0	13	0
Prediction: MCF-10A	0	0	2	20

B

SVM	Actual: MDA-MB-231	Actual: MCF-7	Actual: SK-BR-3	Actual: MCF-10A
Prediction: MDA-MB-231	22	0	1	0
Prediction: MCF-7	1	21	3	0
Prediction: SK-BR-3	0	1	16	0
Prediction: MCF-10A	0	0	2	20

Figure S14. Identification of BC cells with different molecular fingerprints using RT-qPCR. (A) Confusion matrix results based on the LDA model. (B) Confusion matrix results based on the SVM model.

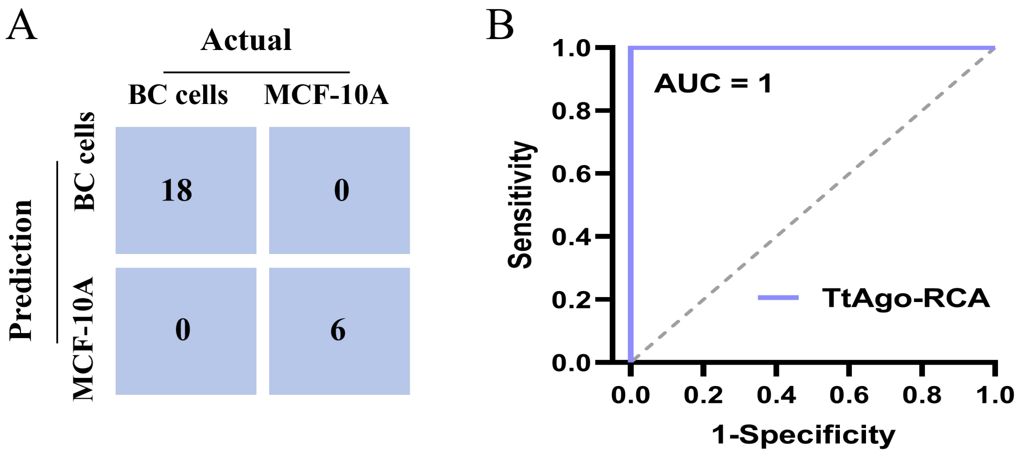

Figure S15. Identification of BC cell lines using TACTIC. (A) Confusion matrix results and (B) ROC analysis.

A

LDA (Prediction \ Actual)	MDA-MB-231	MCF-7	SK-BR-3	MCF-10A
MDA-MB-231	21	0	4	0
MCF-7	1	22	1	0
SK-BR-3	1	0	17	0
MCF-10A	0	0	0	20

B

SVM (Prediction \ Actual)	MDA-MB-231	MCF-7	SK-BR-3	MCF-10A
MDA-MB-231	21	0	4	0
MCF-7	1	21	2	0
SK-BR-3	1	1	16	0
MCF-10A	0	0	0	20

Figure S16. Identification of BC cells with different molecular fingerprints using TACTIC. (A) Confusion matrix results based on the LDA model. (B) Confusion matrix results based on the SVM model.

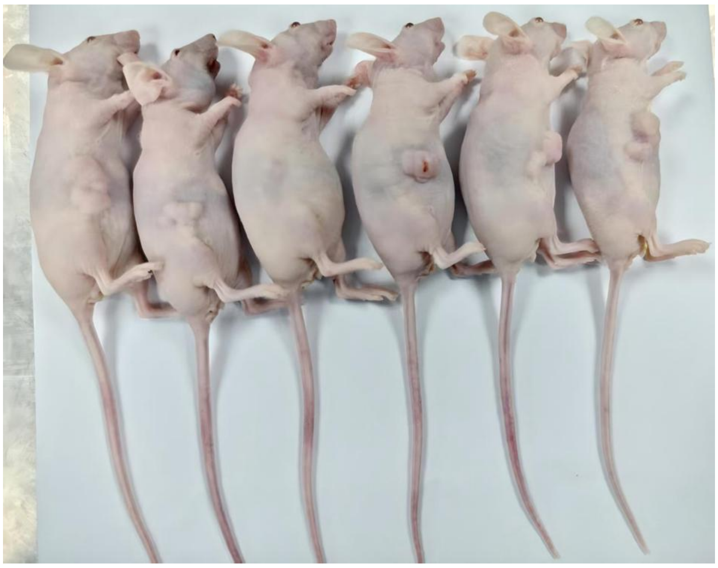

Figure S17. BC-bearing mouse model.

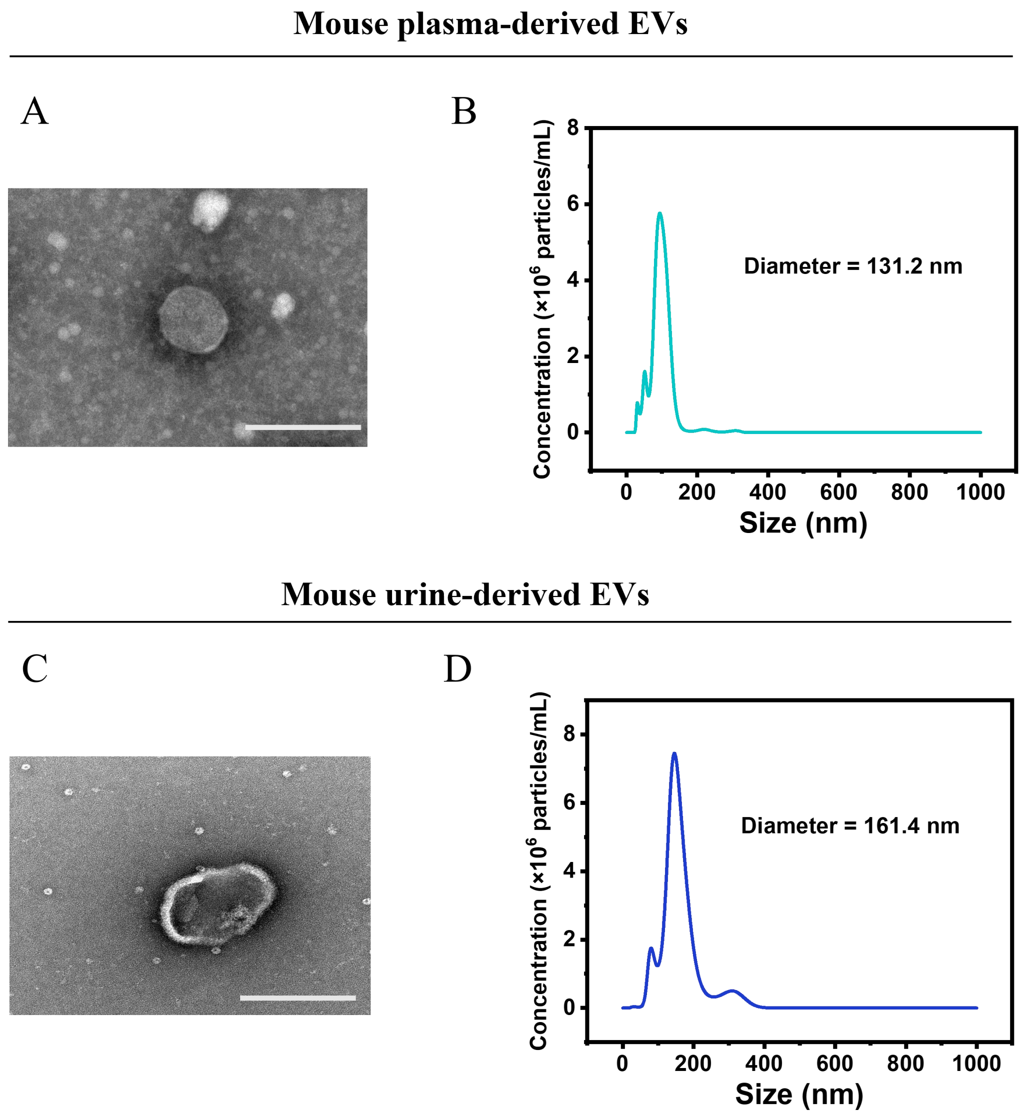

Figure S18. Characterization of EVs from plasma and urine of BC-bearing mice. (A) TEM image and (B) NTA results of mouse plasma-derived EVs; (C) TEM image and (D) NTA results of mouse urine-derived EVs. Scare bar, 200 nm.

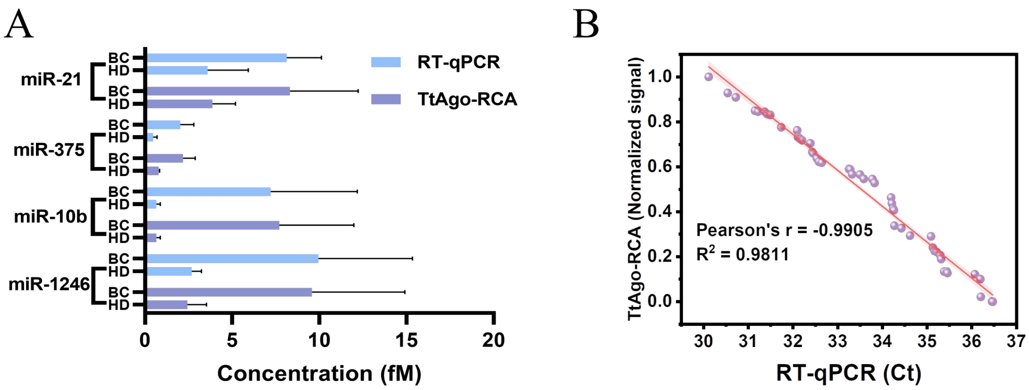

Figure S19. Consistency analysis of two detection methods in mouse plasma samples. (A) Comparison of measured concentrations and (B) linear regression. Bars represent the mean ± SD (n = 6).

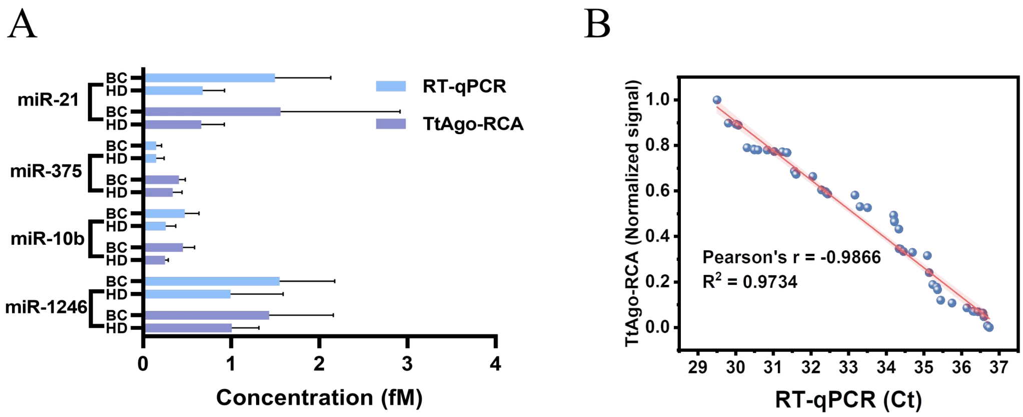

Figure S20. Consistency analysis of two detection methods in mouse urine samples. (A) Comparison of measured concentrations and (B) linear regression. Bars represent the mean ± SD (n = 6).

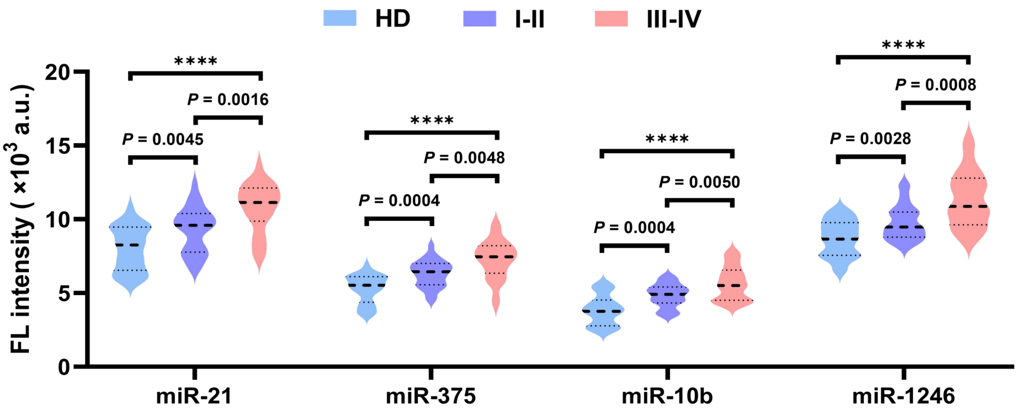

Figure S21. The expression profiles of the four EV miRNAs in different BC stage.

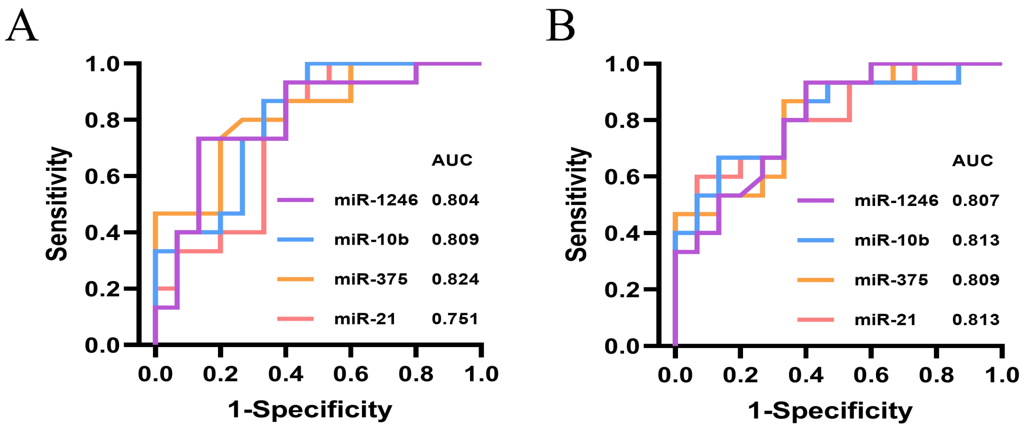

Figure S22. ROC analysis using single marker in 75 clinical cohort. (A) ROC analysis for diagnosing BC in early stage; (B) ROC analysis for diagnosing BC.

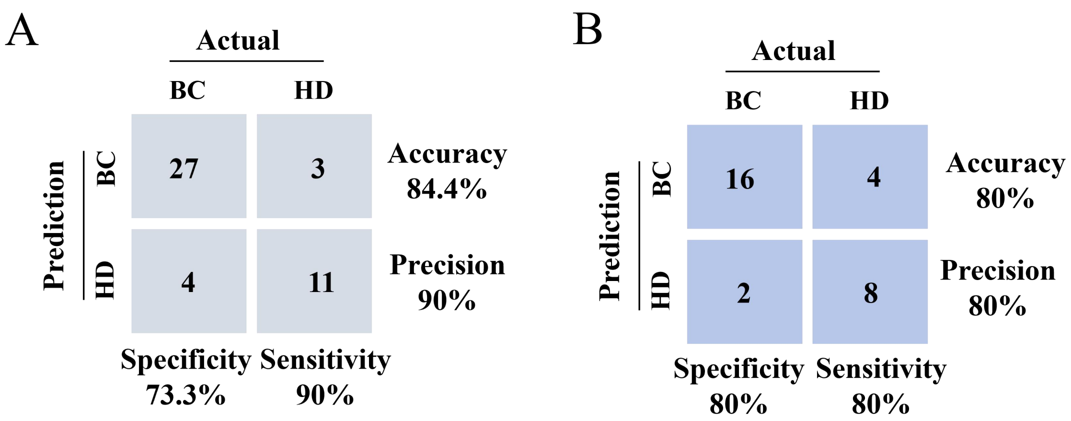

Figure S23. Confusion matrix results in differentiating BC patients from HD. (A) Confusion matrix results in a training cohort; (B) Confusion matrix results in a validation cohort.

Table S1. The sequences used in this work.

Name	Sequence (5'→3')
Padlock 1	P-TAA CCA TCC GGC GCG CGC CGG CTC AAC ATC AGT CTG ATA AGC TAT CGT CGC AGT CCA GCT CAG ATA CCC
BP	GAT GGT TAG GGT ATC TGA dT
miR-21	UAG CUU AUC AGA CUG AUG UUG A
RP	CGG CGC GCG CCG GC
G1	TCG TCG CAG TCC AGC TC
Substrate	GGG TAT CTG AGC TGG ACT GCG ACG ATA GCT TAT CAG ACT GAT GTT GA
Padlock 1-1	P-TAA CCA TCC GGC GCG CGC CGG CAT CGT CAA CAT CAG TCT GAT AAG CTA CGA TTC GTC GCA GTC CAG CTC AGA TAC CC
Padlock 1-2	P-TAA CCA TCC GGC GCG CGC CGG CAT CGT CTG TCA ACA TCA GTC TGA TAA GCT ACA GAC GAT TCG TCG CAG TCC AGC TCA GAT ACC C
Padlock 1-3	P-TAA CCA TCC GGC GCG CGC CGG CAT CGT CTG AGA CTC AAC ATC AGT CTG ATA AGC TAG TCT CAG ACG ATT CGT CGC AGT CCA GCT CAG ATA CCC
Padlock 1-4	P-TAA CCA TCC GGC GCG CGC CGG CAT CGT CTG AGA CTC AAC ATC AGT CTG ATA AGC TAT CGT CGC

	AGT CCA GCT CGT CTC AGA CGA TAG ATA CCC
Padlock 1-5	P-TAA CCA TCC GGC GCG CGC CGG CTC AAC ATC AGT CTG ATA AGC TAA TCG TCT GAG ACT CGT CGC AGT CCA GCT CGT CTC AGA CGA TAG ATA CCC
Padlock 1-6	P-TAA CCA TCC GGC GCG CGC CGG CTC AAC ATC AGT CTG ATA AGC TAG AAT GCT AGA TAC CC
Padlock 1-7	P-TAA CCA TCC GGC GCG CGC CGG CTC AAC ATC AGT CTG ATA AGC TAG CAA TGC CAG ATA CCC
Padlock 1-8	P-TAA CCA TCC GGC GCG CGC CGG CTC AAC ATC AGT CTG ATA AGC TAG CAG TGC CAG ATA CCC
Padlock 1-9	P-CTG ATA AGC TAA GAT ACC CTA ACC ATC CGG CGC GCG CCG GCT CGT CGC AGT CCA GCT CTC AAC ATC AGT
Padlock 2	P-TAA CCA TCC GGC GCG CGC CGG CTC GTC GCA GTC CAG CTC TCA ACA TCA GTC TGA TAA GCT AAG ATA CCC
Padlock 3	P-TAA CCA TCC GGC GCG CGC CGG CTC AAC ATC AGT CTG ATA AGC TAT CGT CGC AGT CCA GCT CTC AAC ATC AGT CTG ATA AGC TAA GAT ACC C
Padlock-375	TAA CCA TCC GGC GCG CGC CGG CTC ACG CGA GCC GAA CGA ACA AAT CGT CGC AGT CCA GCT CAG ATA CCC

miR-375	UUU GUU CGU UCG GCU CGC GUG A
Padlock-10b	P-TAA CCA TCC GGC GCG CGC CGG CCA CAA ATT CGG TTC TAC AGG GTA TCG TCG CAG TCC AGC TCA GAT ACC C
miR-10b	UAC CCU GUA GAA CCG AAU UUG UG
Padlock-1246	P-TAA CCA TCC GGC GCG CGC CGG CCC TGC TCC AAA AAT CCA TTT CGT CGC AGT CCA GCT CAG ATACCC
miR-1246	AAU GGA UUU UUG GAG CAG G
Padlock-KPC	P-TAA CCA TCC GGC GCG CGC CGG CGC GGC GTT ATC ACT GTA TCG TCG CAG TCC AGC TCA GAT ACC C
KPC DNA	GCT GCA ATA CAG TGA TAA CGC CGC CGC CAA TTT G
G2	P-TAT CAC TGT ATT GCA CG
G3	P-CAA ATT GGC GGC GGC GT
Padlock-G12D	P-TAA CCA TCC GGC GCG CGC CGG CTC AGC TCC AAC TAC CAC AAG T**T**T CGT CGC AGT CCA GCT CAG ATA CCC
WT KRAS mRNA	AAC UUG UGG UAG UUG GAG CUG **G**UG GCG UAG GC
KRAS^{G12D} mRNA	AAC UUG UGG UAG UUG GAG CUG **A**UG GCG UAG

	GC
G4	P-TCC TAC GCC ATC AGC T
miR-21-M1	UAG CUU AUG AGA CUG AUG UUG A
miR-21-M2	UAG CUU AUG AGA CUG AUG UUG A
miR-21-M3	UAG CUU AUG AGA CUG UUG UUG A
miR-21-M4	UAG CUU AUG AGA CUG UUC UUG A
miR-375-M1	UUU GUU CGA UCG GCU CGC GUG A
miR-375-M2	UUU GUU CGA UCG CCU CGC GUG A
miR-375-M3	UUU GUU CGU UCG CCU GGC GUG A
miR-375-M4	UUU GUU CGA UCG CCU GGC CUG A
miR-10b-M1	UAC CCU GUU GAA CCG AAU UUG UG
miR-10b-M2	UAC CCU GUU GAA GCG AAU UUG UG
miR-10b-M3	UAC CCU GUU GAA GCG UAU UUG UG
miR-10b-M4	UAC CCU GUU GAA GCG UAU AUG UG
miR-1246-M1	AAU GGA UUA UUG GAG CAG G
miR-1246-M2	AAU GGA UUA UUG CAG CAG G
miR-1246-M3	AAU GGA UUA UUG CAG GAG G
miR-1246-M4	AAA GGA UUA UUG CAG GAG G
miR-429	UAA UAC UGU CUG GUA AAA CCG U
miR-203	GUG AAA UGU UUA GGA CCA CUA G
miR-141	UAA CAC UGU CUG GUA AAG AUG G
let-7a	UGA GGU AGU AGG UUG UAU AGU U

Note: P stands for phosphate group; dT stand for inverted T base; Bold font indicates the TtAgo cutting site; The red font indicates the base mutation site.

Table S2. The sequences used for RT-PCR

Name	Sequence (5'→3')
miR-21 RT primer	GTC GTA TCC AGT GCA GGG TCC GAG GTA TTC GCA CTG GAT ACG ACT CAA CA
miR-21 forward primer	GCG CGT AGC TTA TCA GAC TGA
miR-375 RT primer	GTC GTA TCC AGT GCA GGG TCC GAG GTA TTC GCA CTG GAT ACG ACT CAC GC
miR-375 forward primer	AAC CGG TTT GTT CGT TCG GCT
miR-10b-RT primer	GTC GTA TCC AGT GCA GGG TCC GAG GTA TTC GCA CTG GAT ACG ACC ACA AA
miR-10b forward primer	ACG ACG TAC CCT GTA GAA CCG A
miR-1246 RT primer	GTC GTA TCC AGT GCA GGG TCC GAG GTA TTC GCA CTG GAT ACG ACC CTG CT
miR-1246 forward primer	GCG CGA ATG GAT TTT TGG
Universal reverse primer	ATC CAG TGC AGG GTC CGA GG

Table S3. Comparison with other Ago-based, RCA-based, and endonucleases-based methods for nucleic acid detection

Methods	Biomarkers	Sensitivity	One-pot	Isothermal	Time
TtAgo+EXPAR[1]	miRNA	pM	Yes	No	90 min
NgAgo+TDN[2]	DNA/RNA	fM	No	Yes	2 h
PfAgo+LCR[3]	DNA/RNA	aM	No	Yes	70 min
TtAgo[4]	DNA	fM	Yes	Yes	<2 h
Nb. BbvCI[5]	miRNA	aM	No	Yes	>4 h
DNAzyme[6]	miRNA	pM	Yes	Yes	>2 h
Cas12a+RCA[7]	miRNA	fM	Yes	Yes	<3h
Cas12a+RCA[8]	miRNA	pM	No	Yes	30min
RCA+G4[9]	DNA	pM	NO	Yes	<6 h
TtAgo+RCA (This work)	DNA/RNA	aM	Yes	Yes	30 min

EXPAR: exponential amplification reaction; TDN: tetrahedral DNA nanostructure; LCR: ligase chain reaction; G4: G- Quadruplex

Table S4. Clinical bacteria sample information and analysis results by Minimum Inhibitory Concentration assay (MIC).

Index	Sample type	Carbapenems (mg/L)	Sensitivity	Diagnosis
1	Sputum	≥16	R	KPC
2	Urine	≥16	R	KPC
3	Sputum	≥16	R	KPC
4	Sputum	≥16	R	KPC
5	Sputum	≥16	R	KPC
6	Feces	≥16	R	KPC
7	Urine	≥16	R	KPC
8	Feces	≥16	R	KPC
9	Venous blood	≥16	R	KPC
10	Sputum	≥16	R	KPC
11	Sputum	≥16	R	KPC
12	Sputum	≥16	R	KPC
13	Urine	≥16	R	KPC
14	Urine	≥16	R	KPC
15	Feces	≥16	R	KPC
16	Urine	≥16	R	KPC
17	Sputum	≥16	R	KPC
18	Ascites	≥16	R	KPC

19	Ascites	≥16	R	KPC
20	Sputum	≥16	R	KPC
21	Urine	≤0.25	S	KP
22	Sputum	≤0.25	S	KP
23	Venous blood	≤0.25	S	KP
24	Venous blood	≤0.25	S	KP
25	Sputum	≤0.25	S	KP
26	Sputum	≤0.25	S	KP
27	Sputum	≤0.25	S	KP
28	Venous blood	≤0.25	S	KP
29	Sputum	≤0.25	S	KP
30	Sputum	≤0.25	S	KP
31	Sputum	≤0.25	S	KP
32	Sputum	≤0.25	S	KP
33	Urine	≤0.25	S	KP
34	Sputum	≤0.25	S	KP
35	Sputum	≤0.25	S	KP
36	Sputum	≤0.25	S	KP
37	Sputum	≤0.25	S	KP
38	Venous blood	≤0.25	S	KP
39	Venous blood	≤0.25	S	KP

40	Sputum	≤0.25	S	KP

KPC: Klebsiella pneumoniae carbapenemase; KP: Klebsiella pneumoniae

Table S5. Clinical plasma sample information

Index	Age	TNM stage	Stage number
HD1	58	-	-
HD2	59	-	-
HD3	53	-	-
HD4	68	-	-
HD5	49	-	-
HD6	51	-	-
HD7	45	-	-
HD8	50	-	-
HD9	51	-	-
HD10	51	-	-
HD11	51	-	-
HD12	42	-	-
HD13	68	-	-
HD14	59	-	-
HD15	51	-	-
HD16	42	-	-
HD17	36	-	-

HD18	76	-	-
HD19	40	-	-
HD20	55	-	-
HD21	63	-	-
HD22	69	-	-
HD23	75	-	-
HD24	59	-	-
HD25	62	-	-
HD26	51	-	-
HD27	61	-	-
HD28	67	-	-
HD29	60	-	-
HD30	56	-	-
HD31	61	-	-
HD32	55	-	-
HD33	70	-	-
HD34	60	-	-
HD35	55	-	-
HD36	60	-	-
HD37	35	-	-
HD38	58	-	-

HD39	57	-	-
HD40	65	-	-
BC1	70	T1N0M0	I
BC2	37	T1N3M0	III
BC3	58	T2N0M0	II
BC4	50	T2N0M0	II
BC5	62	T1N1M0	I
BC6	50	T1N0M0	I
BC7	50	T1N0M0	I
BC8	68	T4N1M0	III
BC9	35	T2N2M0	III
BC10	49	T2N0M0	II
BC11	53	T1N1M0	I
BC12	41	T1N0M0	I
BC13	46	T1N0M0	I
BC14	43	T1N0M0	I
BC15	54	T1N0M0	I
BC16	49	T1N1M0	I
BC17	40	T2N1M0	II
BC18	58	T1N1M0	I
BC19	58	T1N0M0	I

BC20	49	T2N0M0	II
BC21	65	T1N0M0	I
BC22	60	T2N1M0	II
BC23	39	T2N1M0	II
BC24	42	T2N1M0	II
BC25	51	T2N0M0	II
BC26	52	T2N0M0	II
BC27	48	T2N1M0	II
BC28	39	T2N1M0	II
BC29	57	T2N0M0	II
BC30	60	T1N1M0	I
BC31	42	T1N0M0	I
BC32	47	T1N0M0	I
BC33	45	T1N1M0	I
BC34	40	T2N1M0	II
BC35	56	T2N0M0	II
BC36	58	T1N1M0	I
BC37	55	T1N1M0	I
BC38	46	T1N0M0	I
BC39	71	T1N1M0	I
BC40	41	T1N0M0	I

BC41	40	T4N1M1	IV
BC42	58	T4N1M1	IV
BC43	57	T2N2M0	III
BC44	51	T2N2M1	IV
BC45	76	T4N0M0	III
BC46	43	T4N1M0	III
BC47	55	T4N1M0	III
BC48	56	T2N1M1	IV
BC49	38	T2N2M0	III
BC50	62	T2N3M0	III
BC51	54	T3N1M0	III
BC52	68	T1N2M0	III
BC53	43	T2N2M0	III
BC54	69	T4N1M0	III
BC55	54	T4N1M0	III
BC56	48	T2N2M0	III
BC57	35	T2N2M0	III
BC58	30	T4N1M1	IV
BC59	47	T1N0M1	IV
BC60	58	T4N1M1	IV
BC61	46	T4N1M1	IV

BC62	52	T4N1M1	IV
BC63	55	T4N1M1	IV
BC64	59	T2N3M0	III
BC65	35	T3N2M0	III

The TNM staging system, maintained by the American Joint Committee on Cancer (AJCC) and endorsed by the Union for International Cancer Control (UICC), is utilized to describe the extent and severity of cancer. In this system, T (Tumor) indicates the size and extent of the primary tumor, N (Nodes) denotes whether the tumor has spread to regional lymph nodes, and M (Metastasis) refers to whether the tumor has metastasized to distant parts of the body. The overall stage of breast cancer, ranging from stage 0 to stage IV, is determined by a combination of the T, N, and M categories.

Reference

[1] Q. Lin, Y. Huang, J. Chen, Z. Lei, M. Yang, M. Kudouluke, G. Su, J. Chen, *Anal. Chem.* 2025, 97 (7), 3981.

[2] B. Yang, H. Wang, J. Kong, X. Fang, *Nat. Commun.* 2024, 15 (1).

[3] L. Wang, R. He, B. Lv, X. Yu, Y. Liu, J. Yang, W. Li, Y. Wang, H. Zhang, G. Yan, W. Mao, L. Liu, F. Wang, L. Ma, *Talanta* 2021, 227.

[4] H. Jang, J. Song, S. Kim, J.-H. Byun, K. G. Lee, K.-H. Park, E. Woo, E.-K. Lim, J. Jung, T. Kang, *Nat. Commun.* 2023, 14 (1).

[5] S. Yu, S. Chen, Y. Dang, Y. Zhou, J.-J. Zhu, *Anal. Chem.* 2022, 94 (41), 14349.

[6] X. Huang, Z. Li, Y. Tong, Y. Zhang, T. Shen, M. Chen, Z. Huang, Y. Shi, S. Wen, S.-Y. Liu, J. Guo, X. Zou, Z. Dai, *Anal. Chem.* 2023, 95 (31), 11793.

[7] H. Yan, Y. Wen, Z. Tian, N. Hart, S. Han, S. J. Hughes, Y. Zeng, *Nat. Biomed. Eng.* 2023, 7 (12), 1583.

[8] X. Shen, Z. Lin, X. Jiang, X. Zhu, S. Zeng, S. Cai, H. Liu, *Biosens. Bioelectron.* 2024, 264.

[9] X. Xie, X. Cheng, J. Dong, J. Li, L. Jiang, T. Yang, B. Liao, S. Ding, Q. Liu, F. Luo, W. Cheng, J. Chen, *Anal. Chem.* 2023, 95 (5), 3098.
